# Supplementary material for: Light-induced fine-tuning of optical cavities for organic optoelectronic devices
Source: Nat Commun. 2025 Sep 25;16:8426. doi: 10.1038/s41467-025-64272-7 (PMC12462449; doi:10.1038/s41467-025-64272-7)
Supplement: Supplementary file 1 — Supplementary Information [file 41467_2025_64272_MOESM1_ESM.pdf]

Supplementary Information

Light-induced fine-tuning of optical cavities for organic optoelectronic devices

Shen Xing[1], Eva Bittrich[2], Vasiliki Prifti[1], Stephanie Buchholtz[1], Yuan Liu[3], Louis Conrad Winkler[1], Maximilian F. X. Dorfner[4], Mikhail Malanin[2], Mingchao Wang[5], Guoqin Liu[6], Dinara Samigullina[1], Anna-Lena Hofmann[1], Jakob Wolansky[1], Jörn Vahland[1], Tianyi Zhang[1], Rongjuan Huang[1], Samuel Dominic Seddon[7], Dieter Fischer[2], Sebastian Reineke[1], Frank Ortmann[4], Xinliang Feng[6], Hans Kleemann[1*], Johannes Benduhn[1*], and Karl Leo[1*]*

[1]Dresden Integrated Center for Applied Physics and Photonic Materials (IAPP) and Institute of Applied Physics, Technische Universität Dresden, Nöthnitzer Straße 61, 01187 Dresden, Germany

[2]Leibniz-Institut für Polymerforschung Dresden e.V., Division of Macromolecular Chemistry, Hohe Straße 6, 01069 Dresden, Germany

[3]Key Laboratory of the Ministry of Education for Optoelectronic Measurement Technology and Instrument, Beijing Information Science & Technology University, No. 12 Xiaoying East Road, Beijing, 100192 China.

[4]Department of Chemistry, TUM School of Natural Sciences and Atomistic Modeling Center, Munich Data Science Institute, Technische Universität München, 85748 Garching b. München, Germany.

[5]State Key Laboratory of Advanced Waterproof Materials, School of Advanced Materials, Peking University, Shenzhen Graduate School, Shenzhen 518055, China

[6]Faculty of Chemistry and Food Chemistry, Technische Universität Dresden, Bergstraße 66, 01069 Dresden, Germany.

[7]Institute of Applied Physics, Technische Universität Dresden, Nöthnitzer Straße 61, 01187 Dresden, Germany

Corresponding authors: shen.xing@tu-dresden.de, johannes.benduhn@tu-dresden.de, hans.kleemann1@tu-dresden.de, and karl.leo@tu-dresden.de.

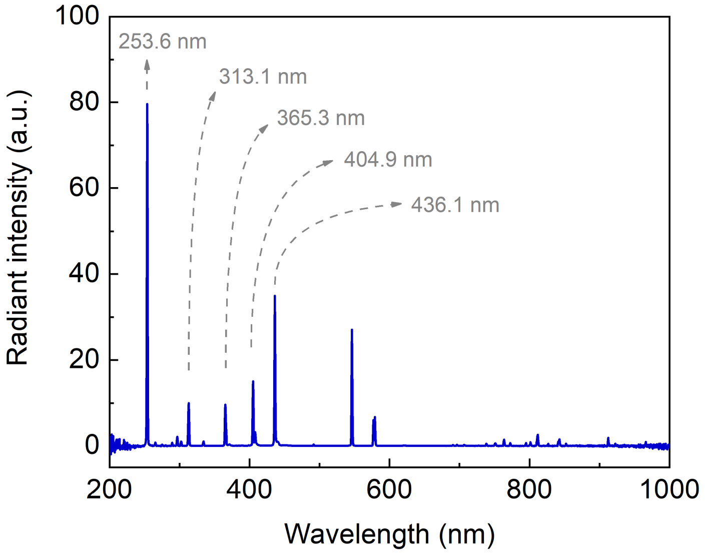

Supplementary Fig. 1. UV amalgam lamp spectrum. Radiant power (a.u.) of employed UV amalgam lamp as a function of wavelength (nm). The UVC irradiance is 250 W m^{-2}.

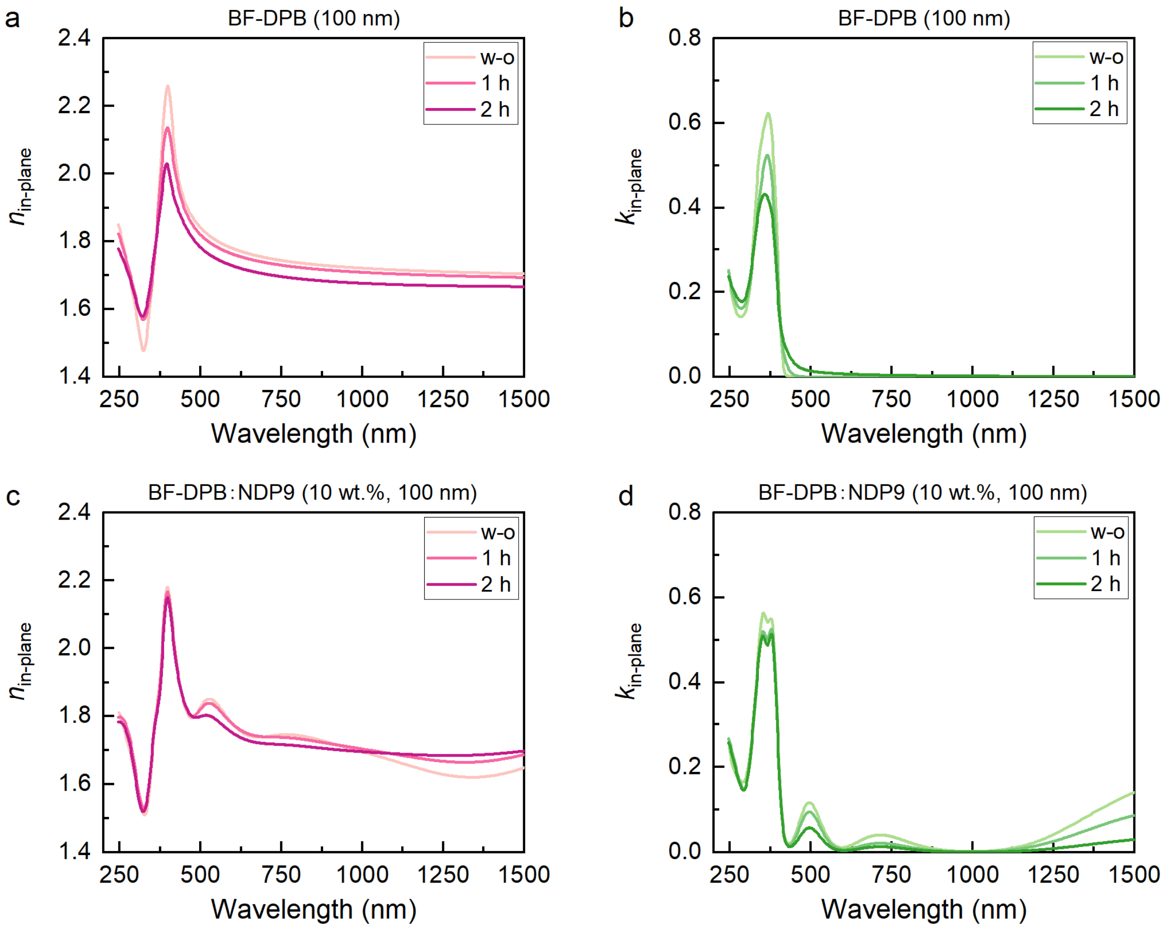

Supplementary Fig. 2. Optical properties of UV-irradiated organic HTLs. Refraction index (*n*) and extinction coefficient (*k*) of (**a**, **b**) BF-DPB (100 nm) and (**c**, **d**) BF-DPB:NDP9 (10 wt.%, 100 nm) under different UV irradiation time.

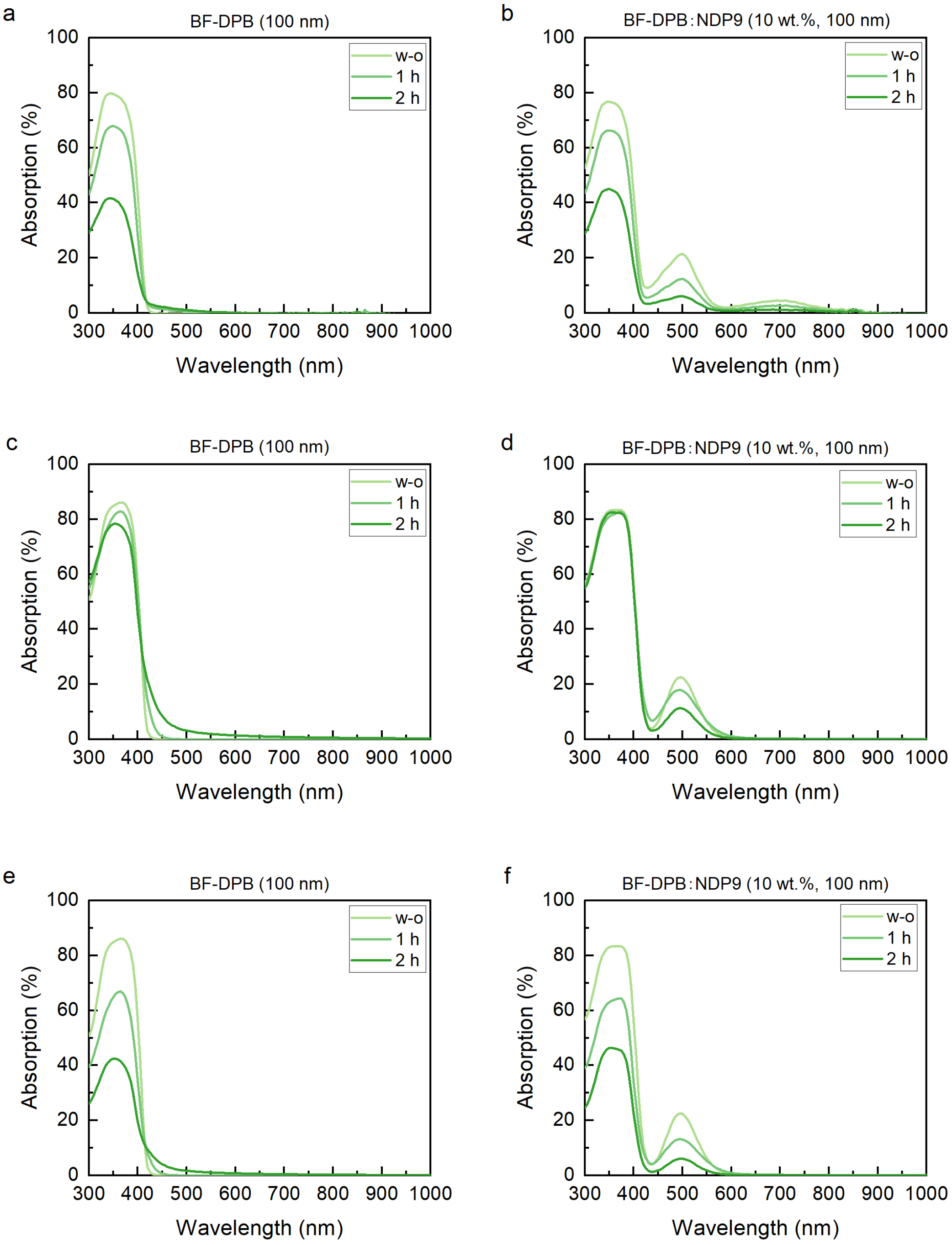

Supplementary Fig. 3. Comparison of absorption spectra. Measured absorption spectra of (**a**) BF-DPB and (**b**) BF-DPB:NDP9 on quartz substrates under different UV irradiation time. The initial thickness of both films is 100 nm. Simulated absorption spectra of (**c**) and **(e)** BF-DPB, and (**d**) and **(f)** BF-DPB:NDP9 on quartz substrates based on the n/k data provided in Supplementary Fig. 2. **(c)** and **(d)** are modeled with a fixed thickness of 100 nm, while **(e)** and **(f)** are simulated with reduced thicknesses based on the reduction rate shown in Case B of Supplementary Table 1.

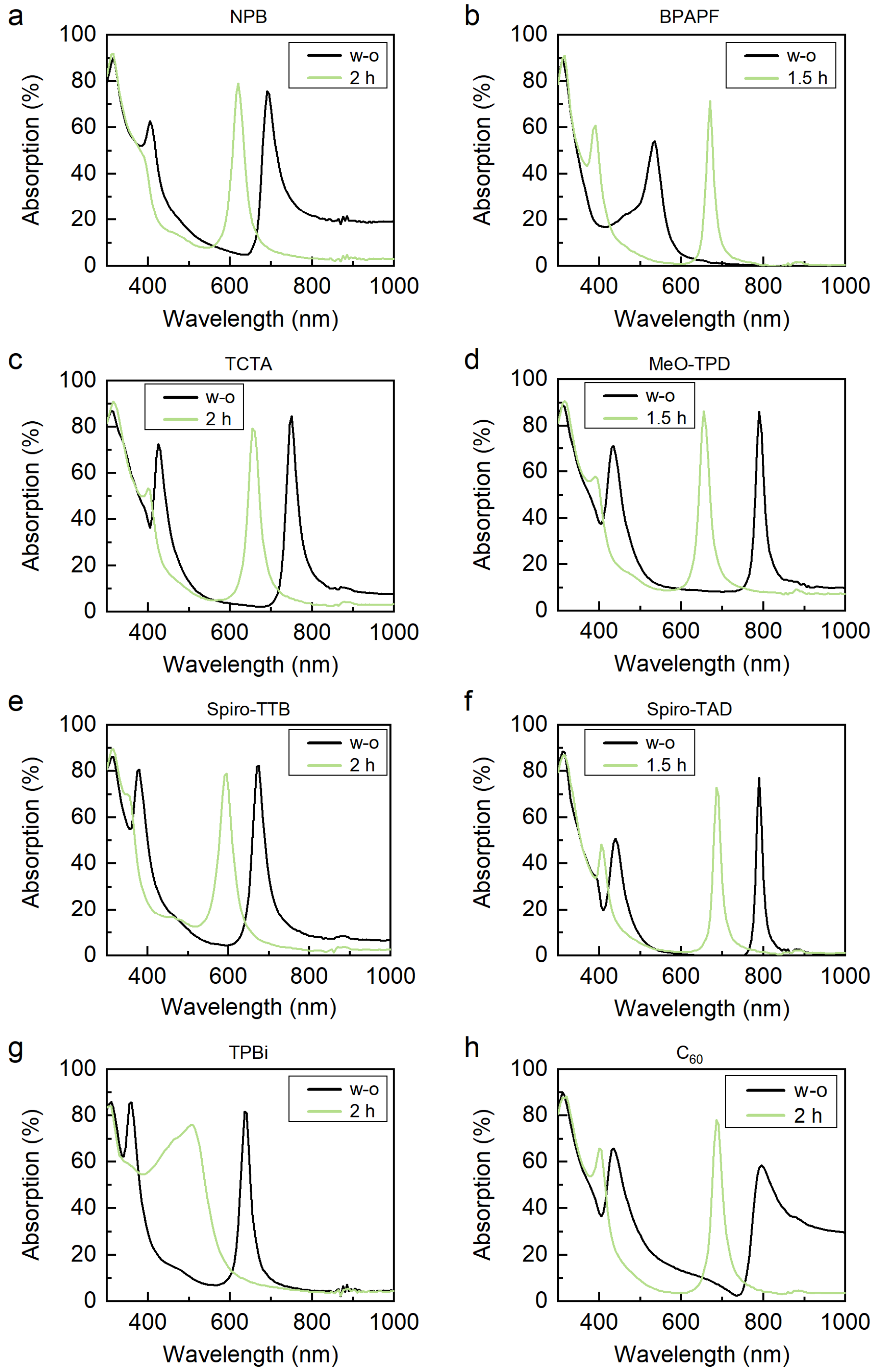

Supplementary Fig. 4. Thickness thinning effect on various organic materials. Absorption spectra of test organic materials after 2 h of UV (**a, c, e, g, h**) or 1.5 h of UV (**b, d, f**) irradiation within a Cr (3 nm) / Au (60 nm) / organic layer (150 nm) / Ag (25 nm) cavity structure. The organic materials involve (**a**) NPB, (**b**) BPAPF, (**c**) TCTA, (**d**) MeO-TPD, (**e**) Spiro-TTB, (**f**) Spiro-TAD, (**g**) TPBi, and (**h**) C_{60}.

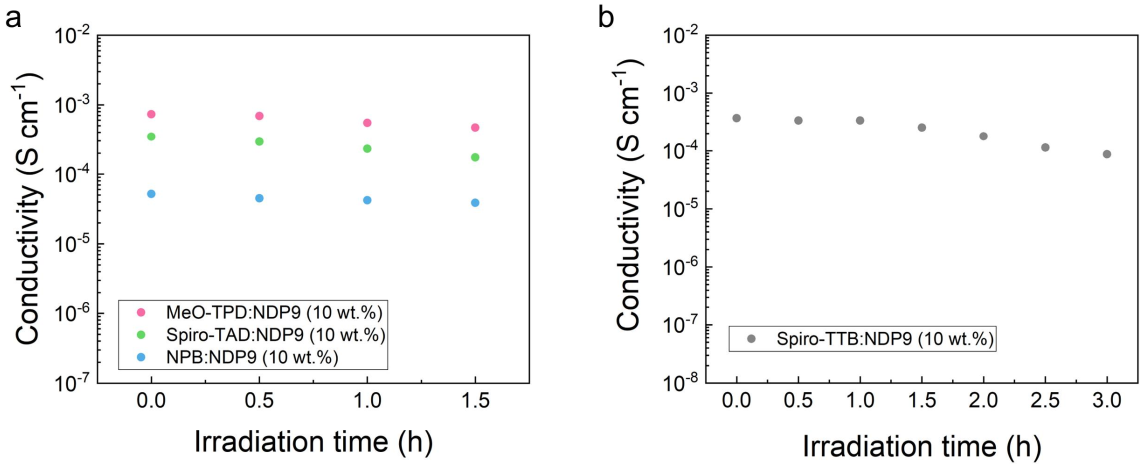

Supplementary Fig. 5. Conductivity of UV-irradiated NDP9-doped organic materials. (**a**) Conductivity of MeO-TPD:NDP9, Spiro-TAD:NDP9, and NPB:NDP9 (10 wt.%) as a function of UV irradiation time. (**b**) Conductivity of Spiro-TTB:NDP9 (10 wt.%) under prolonged UV exposure.

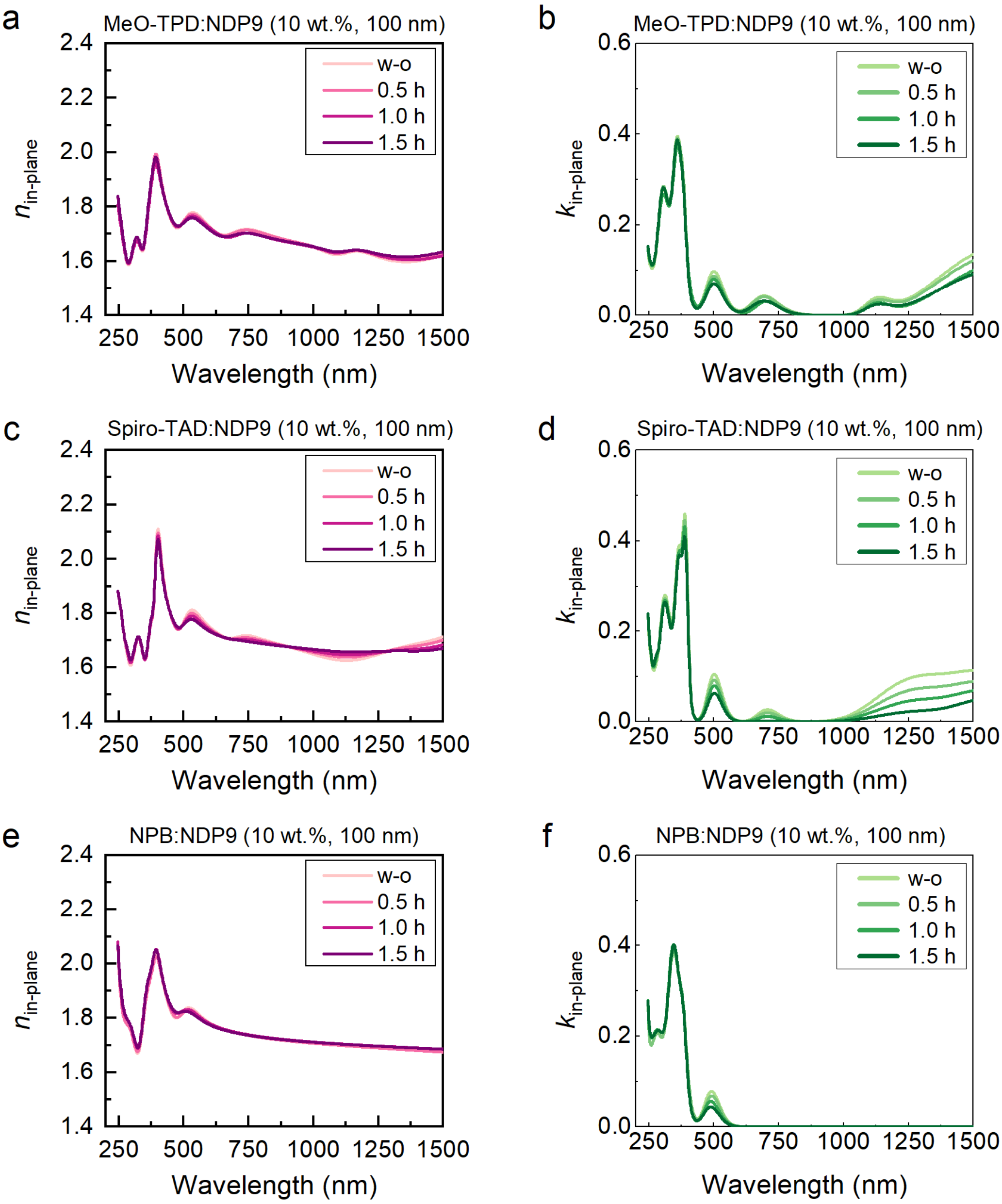

Supplementary Fig. 6. Optical properties of UV-irradiated NDP9-doped organic materials. Refraction index (*n*) and extinction coefficient (*k*) of (**a, b**) MeO-TPD:NDP9 (10 wt.%, 100 nm), (**c, d**) Spiro-TAD:NDP9 (10 wt.%, 100 nm) and (**e, f**) NPB:NDP9 (10 wt.%, 100 nm) under different UV irradiation time.

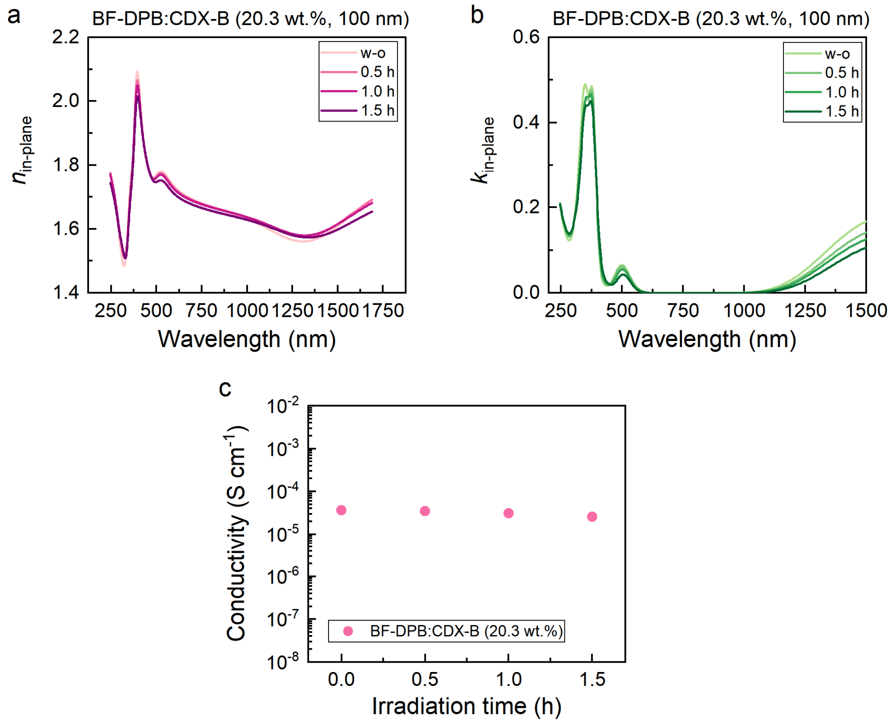

Supplementary Fig. 7. Optical and electrical properties of BF-DPB:CDX-B film (20.3 wt.%, 100 nm). (**a**) Refractive index (*n*), (**b**) extinction coefficient (*k*), and (**c**) conductivity under UV irradiation.

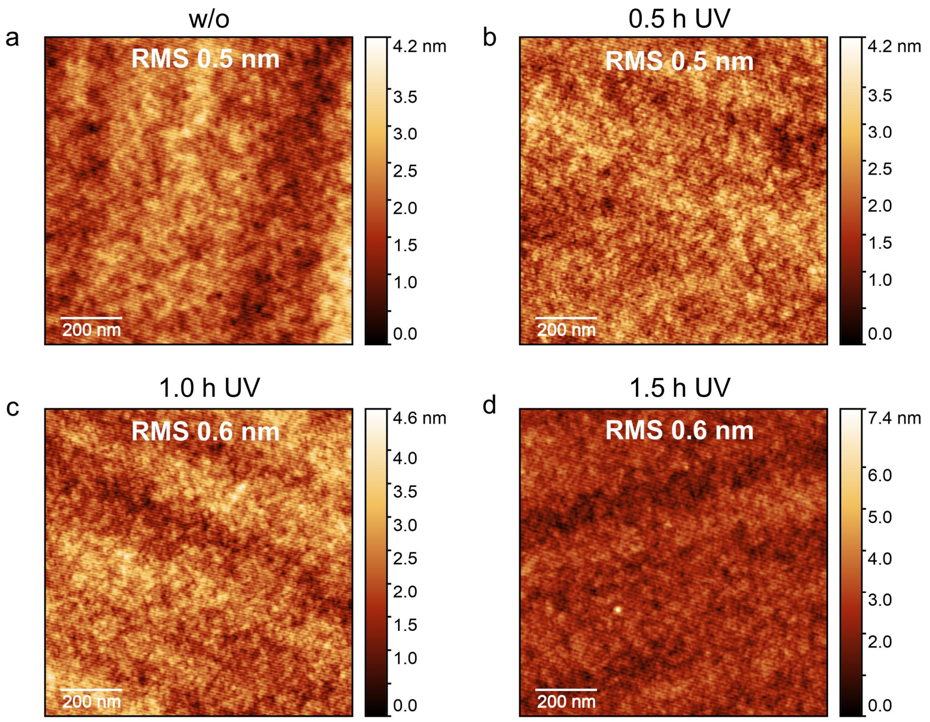

Supplementary Fig. 8. AFM images of BF-DPB:NDP9 films (10 wt.%, 100 nm). (**a**) Before and after UV irradiation for (**b**) 0.5 h, (**c**) 1.0 h, and (**d**) 1.5 h.

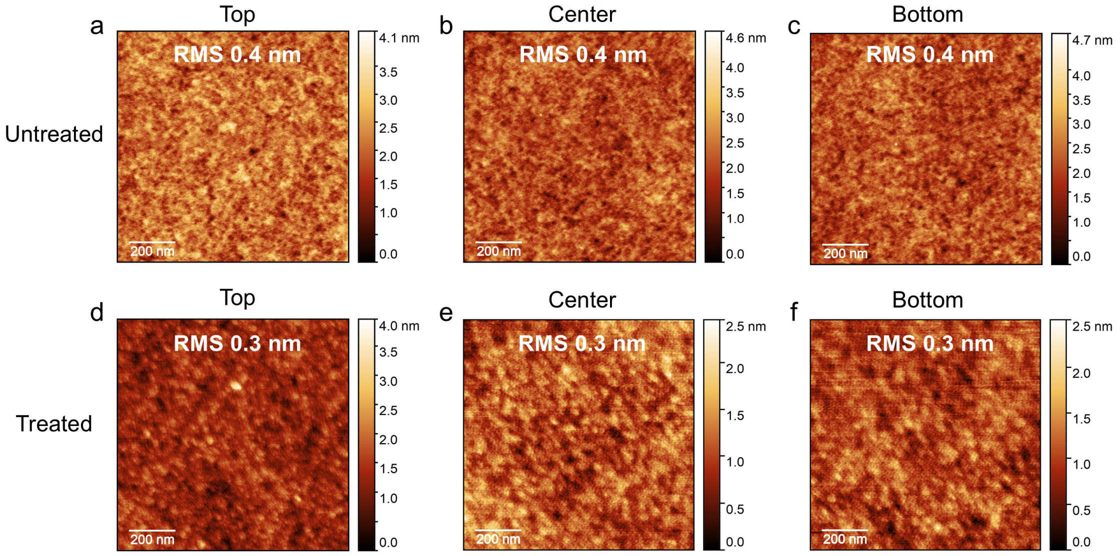

Supplementary Fig. 9. AFM images of BF-DPB:NDP9 films (10 wt.%, 50 nm) deposited on a large-area substrate (15 cm × 15 cm). (**a-c**) Top, center, and bottom positions of the film before UV treatment; (**d-f**) corresponding positions after 0.5 h of UV treatment.

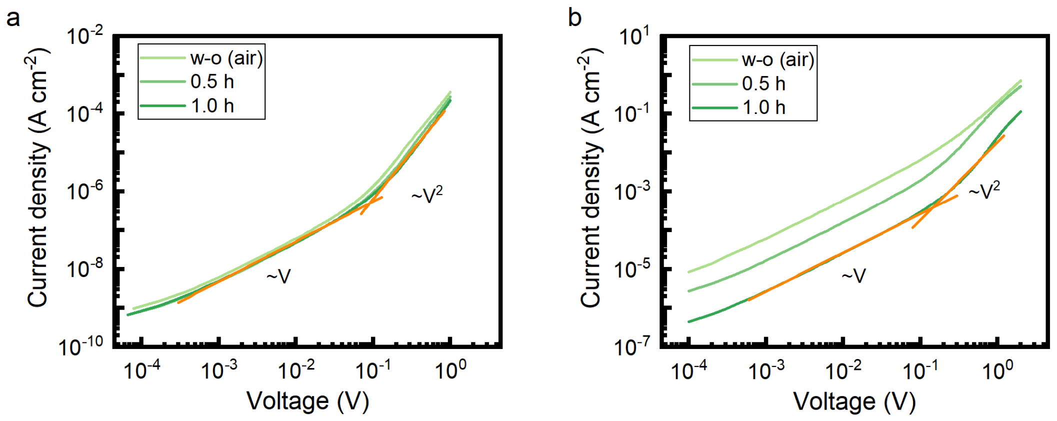

Supplementary Fig. 10. Space charge-limited current (SCLC) measurements of hole-only and electron-only devices under UV treatment. (**a**) Hole mobility of BF-DPB. (**b**) Electron mobility of C_{60}. The hole-only device architecture is ITO / MoO_3 (5 nm) / BF-DPB (120 nm) / MoO_3 (5 nm) / Ag (100 nm). The electron-only device architecture is ITO / BPhen:Cs (1:1, 5 nm) / C_{60} (120 nm) / LiF (1 nm) / Al (100 nm).

To evaluate intrinsic material stability under UV treatment in air, carrier mobility in BF-DPB and C_{60} films is measured using the SCLC method. For BF-DPB, the hole mobility of air-exposed, untreated films is 4.38×10^{-5} cm^2/(V·s), closely matching the vacuum value of 5.7×10^{-5} cm^2/(V·s). After 1 h of UV exposure in ambient conditions, the mobility decreases moderately to 2.16×10^{-5} cm^2/(V·s), suggesting that charge transport pathways remain largely intact. In the case of C_{60}, electron mobility decreases from 6.32×10^{-3} cm^2/(V·s) (1 h in air) to 1.94×10^{-3} cm^2/(V·s) after 1 h of UV treatment. While some degradation is observed, the mobility remains within a functional range. These results confirm that intrinsic materials exhibit moderate UV–air sensitivity, whereas doped systems demonstrate improved electrical stability. Strategies such as reduced UV exposure via laser processing or treatment in controlled N_2/O_2 atmospheres may further mitigate degradation.

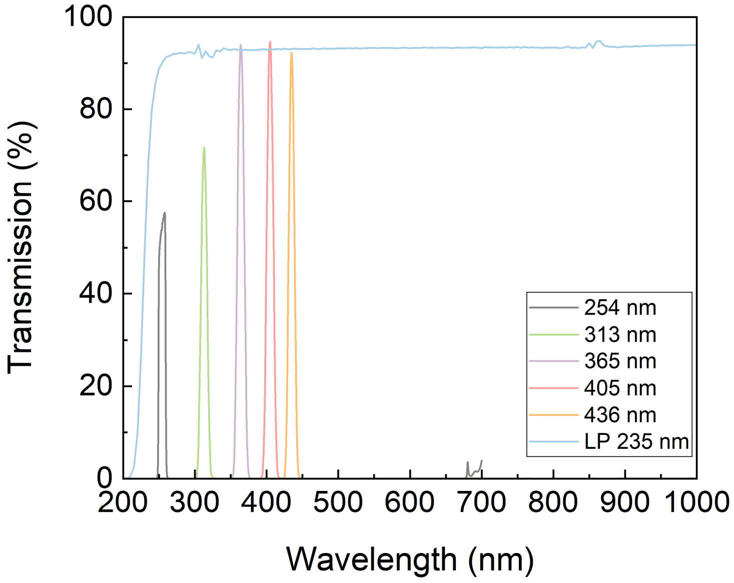

Supplementary Fig. 11. Transmission spectra of employed optical bandpass filters and the LP filter at 235 nm.

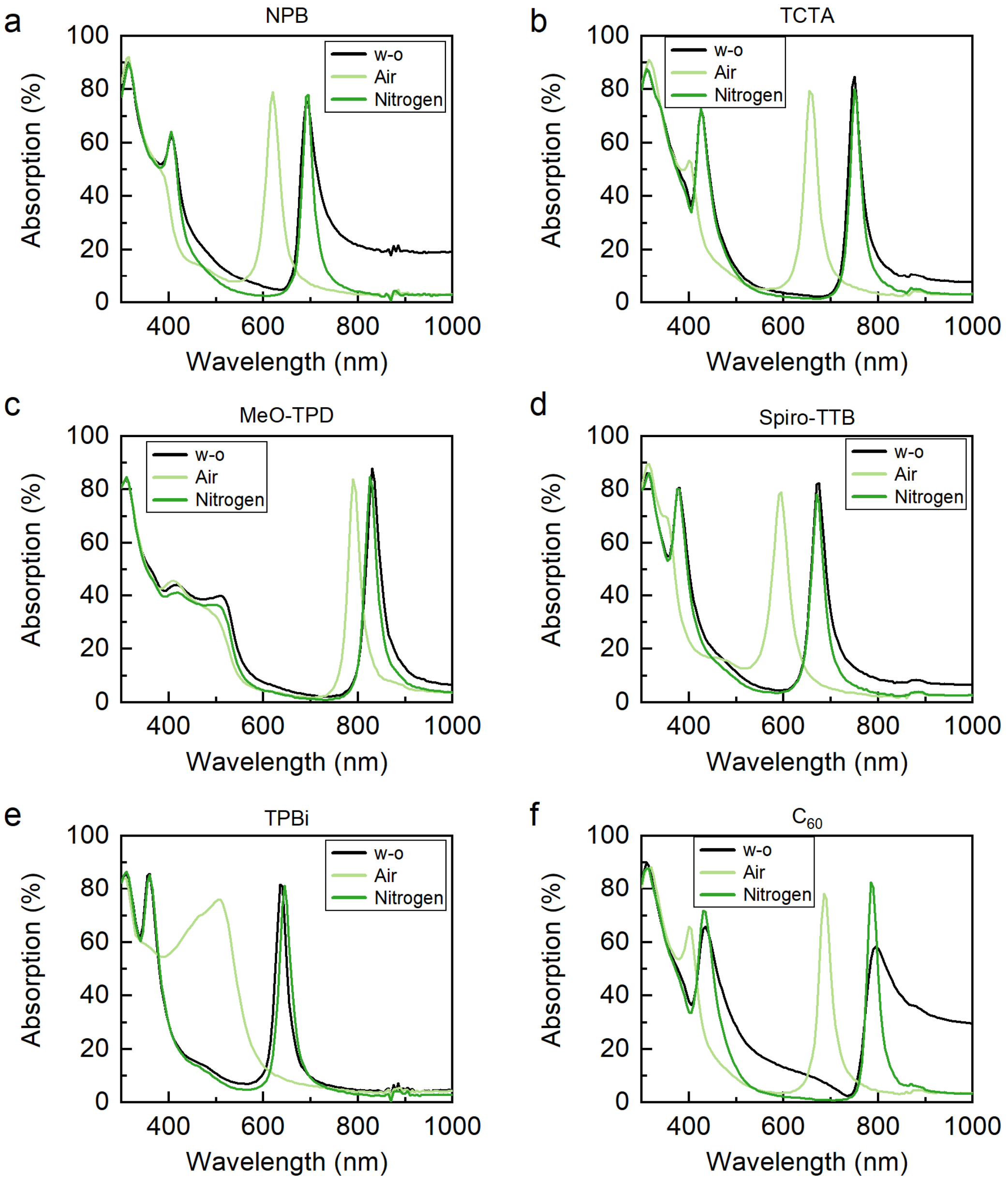

Supplementary Fig. 12. Investigation of the treatment atmosphere on various organic materials under UV irradiation. Absorption spectra of different organic materials after 2 h of UV irradiation in nitrogen and air atmosphere within a Cr (3 nm) / Au (60 nm) / organic layer (150 nm) / Ag (25 nm) cavity structure. The organic materials involve (**a**) NPB, (**b**) TCTA, (**c**) MeO-TPD, (**d**) Spiro-TTB, (**e**) TPBi, and (**f**) C_{60}.

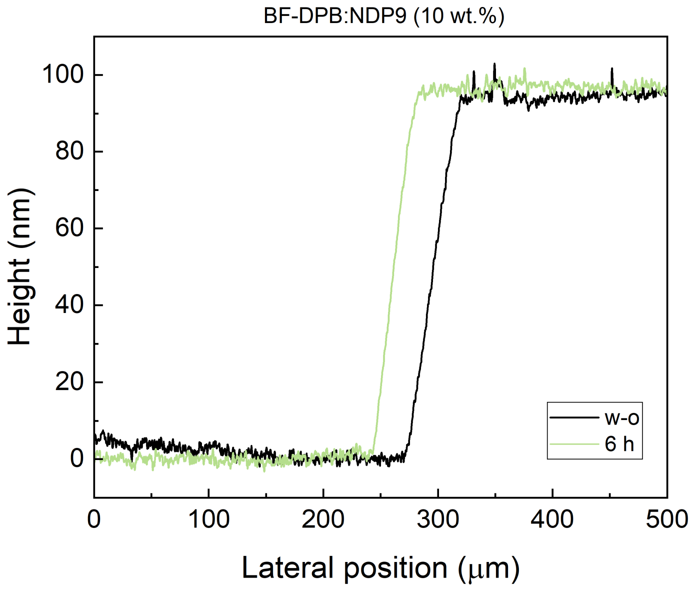

Supplementary Fig. 13. Investigation of the combined effects of air and ozone on the film thinning. Thickness of BF-DPB:NDP9 (10 wt.%) film before and after 6 h in an atmosphere of air and ozone.

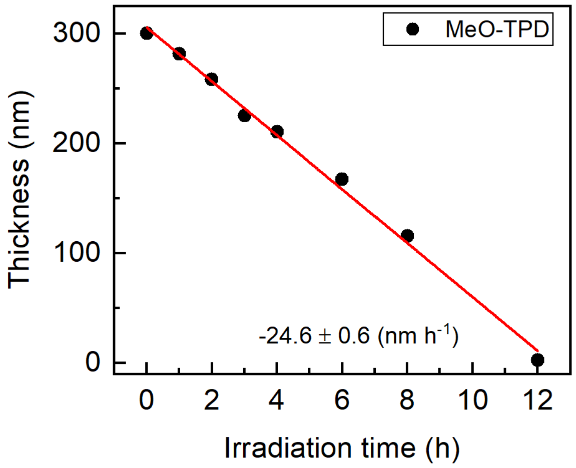

Supplementary Fig. 14. Thickness variation of UV-irradiated MeO-TPD film. The rate of the thickness reduction is approximately 24.6 nm h^{-1}, closely mirroring that of BF-DPB.

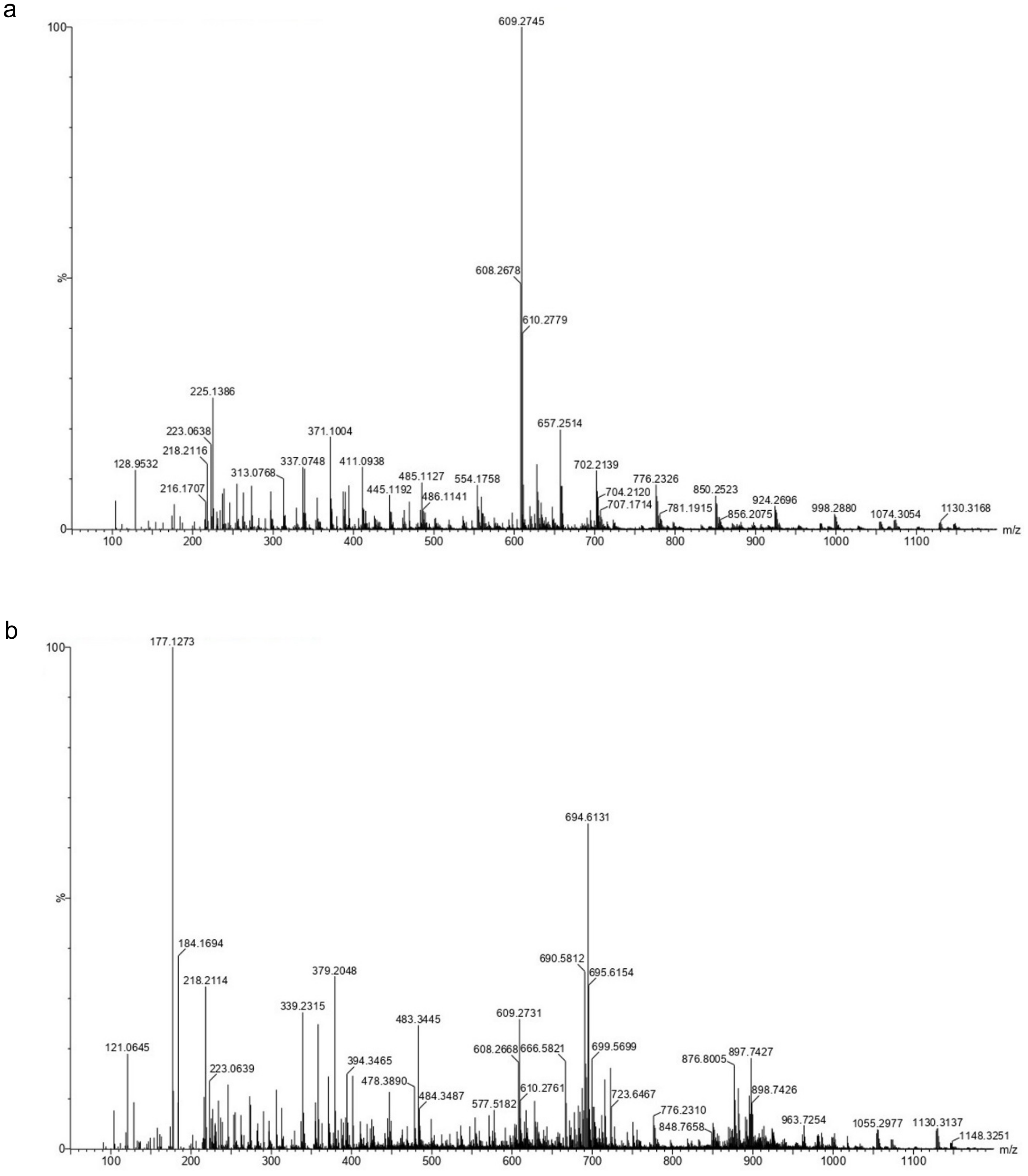

Supplementary Fig. 15. HPLC-MS spectrum of MeO-TPD film. (**a**) Before and (**b**) after 3 h of UV irradiation. The initial thickness of film is 150 nm.

Identified main isomers:

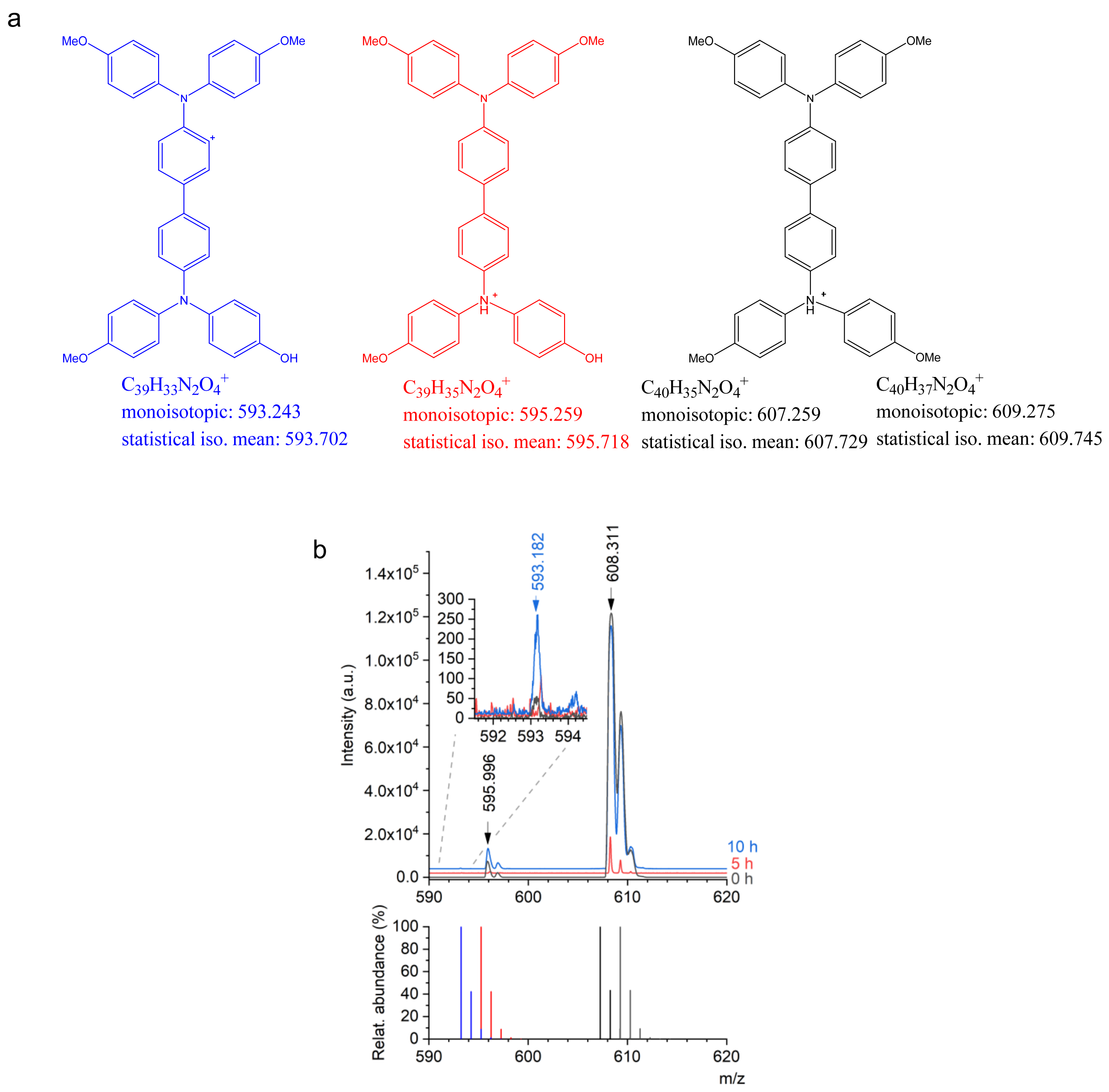

Supplementary Fig. 16-1. MALDI-TOF analysis of chemical structures. Chemical structures (**a**) of the identified main isomers that yield the measured m/z (**b**) of MeO-TPD irradiated by UV for 0, 5 and 10 h, compared to the theoretical isotopic patterns of identified chemical structures shown in (a).

Ether-linked dimers and isomers:

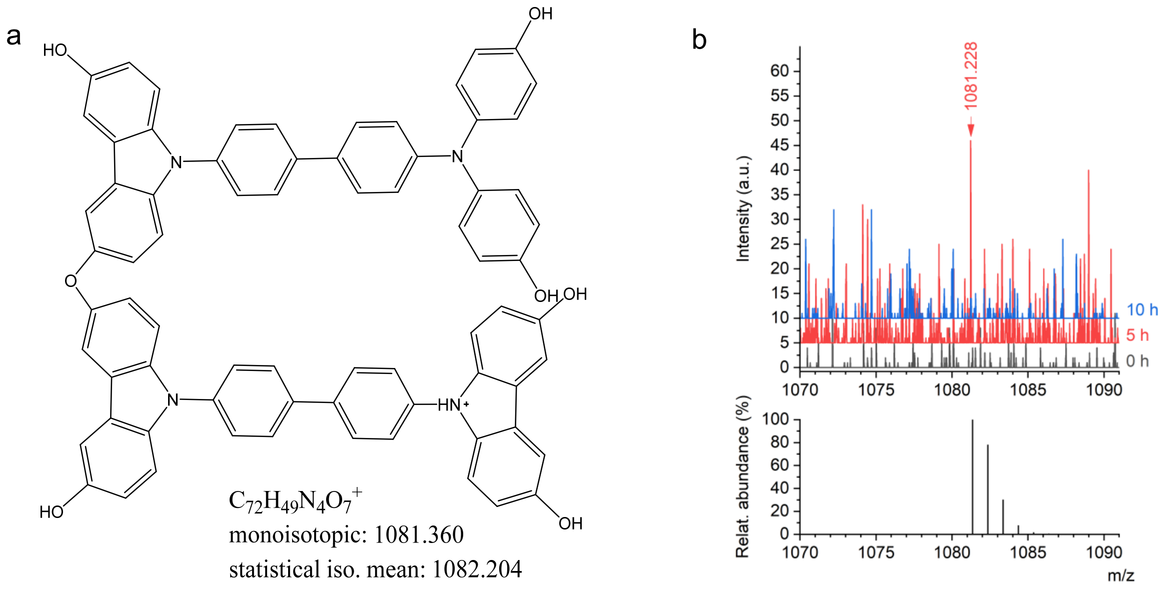

Supplementary Fig. 16-2. MALDI-TOF analysis of chemical structures. Chemical structures (**a**) of the identified Ether-linked dimers that yield the measured m/z (**b**) of MeO-TPD irradiated by UV for 0, 5 and 10 h, compared to the theoretical isotopic patterns of identified chemical structures shown in (a).

Oligomers

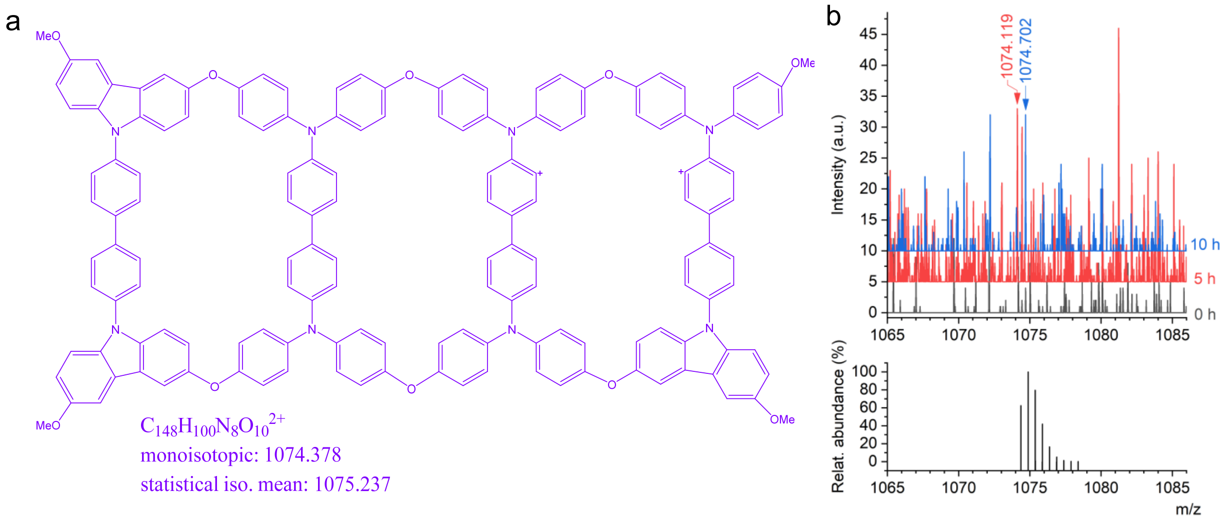

Supplementary Fig. 16-3. MALDI-TOF analysis of chemical structures. Chemical structures (**a**) of the identified oligomer of equal m/z that yield the measured m/z (**b**) of MeO-TPD irradiated by UV for 0, 5 and 10 h, compared to the theoretical isotopic patterns of identified chemical structures shown in (a).

Fragments and cross-reaction products:

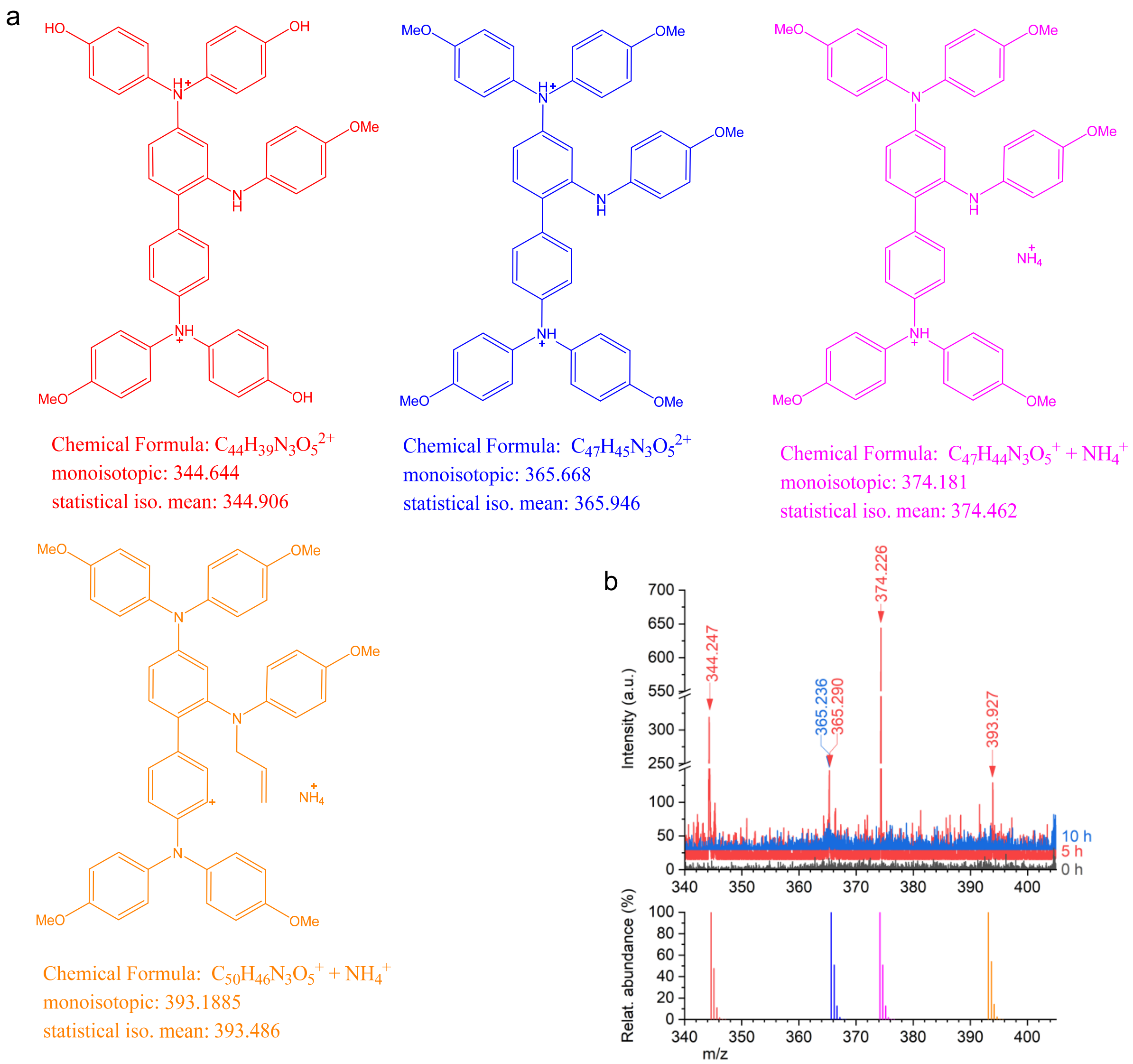

Supplementary Fig. 16-4. MALDI-TOF analysis of chemical structures. Chemical structures (**a**) of identified fragments that yield the measured m/z (**b**) of MeO-TPD irradiated by UV for 0, 5 and 10 h, compared to the theoretical isotopic patterns of identified chemical structures shown in (a).

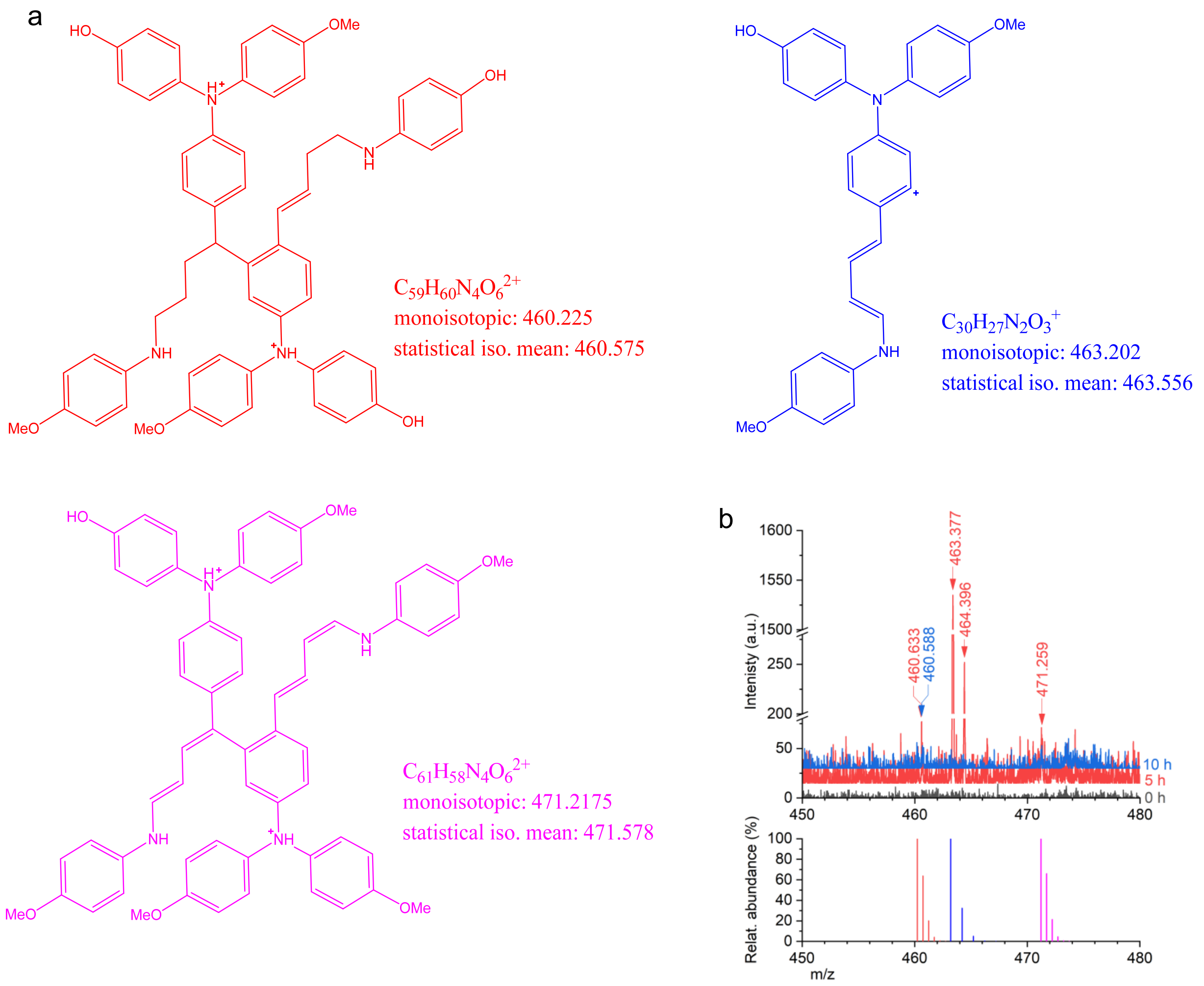

Supplementary Fig. 16-5. MALDI-TOF analysis of chemical structures. Chemical structures (**a**) of identified fragments that yield the measured m/z (**b**) of MeO-TPD irradiated by UV for 0, 5 and 10 h, compared to the theoretical isotopic patterns of identified chemical structures shown in (a).

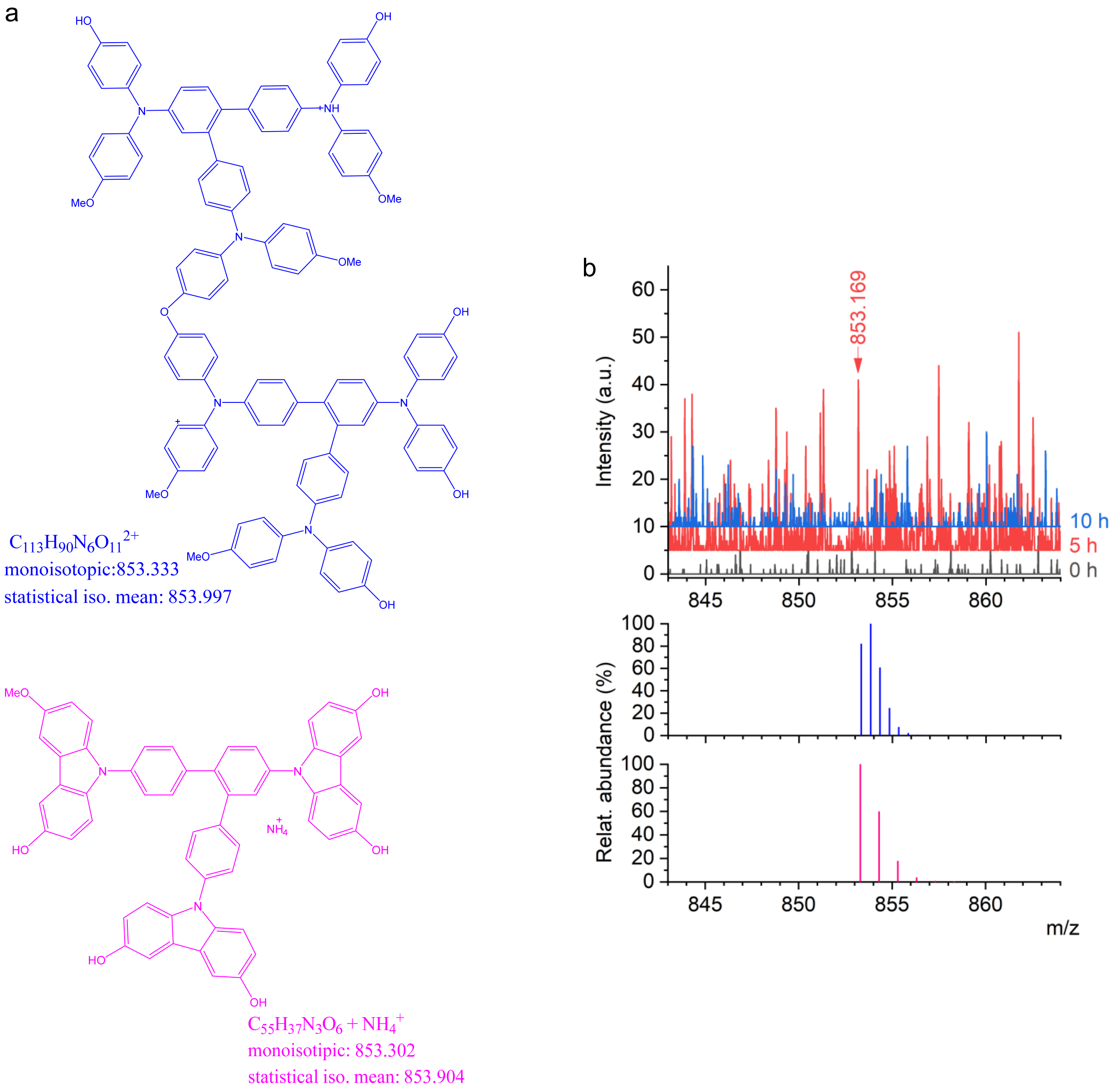

Supplementary Fig. 16-6. MALDI-TOF analysis of chemical structures. Chemical structures (**a**) of the cross-reaction products that yield the measured m/z (**b**) of MeO-TPD irradiated by UV for 0, 5 and 10 h, compared to the theoretical isotopic patterns of identified chemical structures shown in (a).

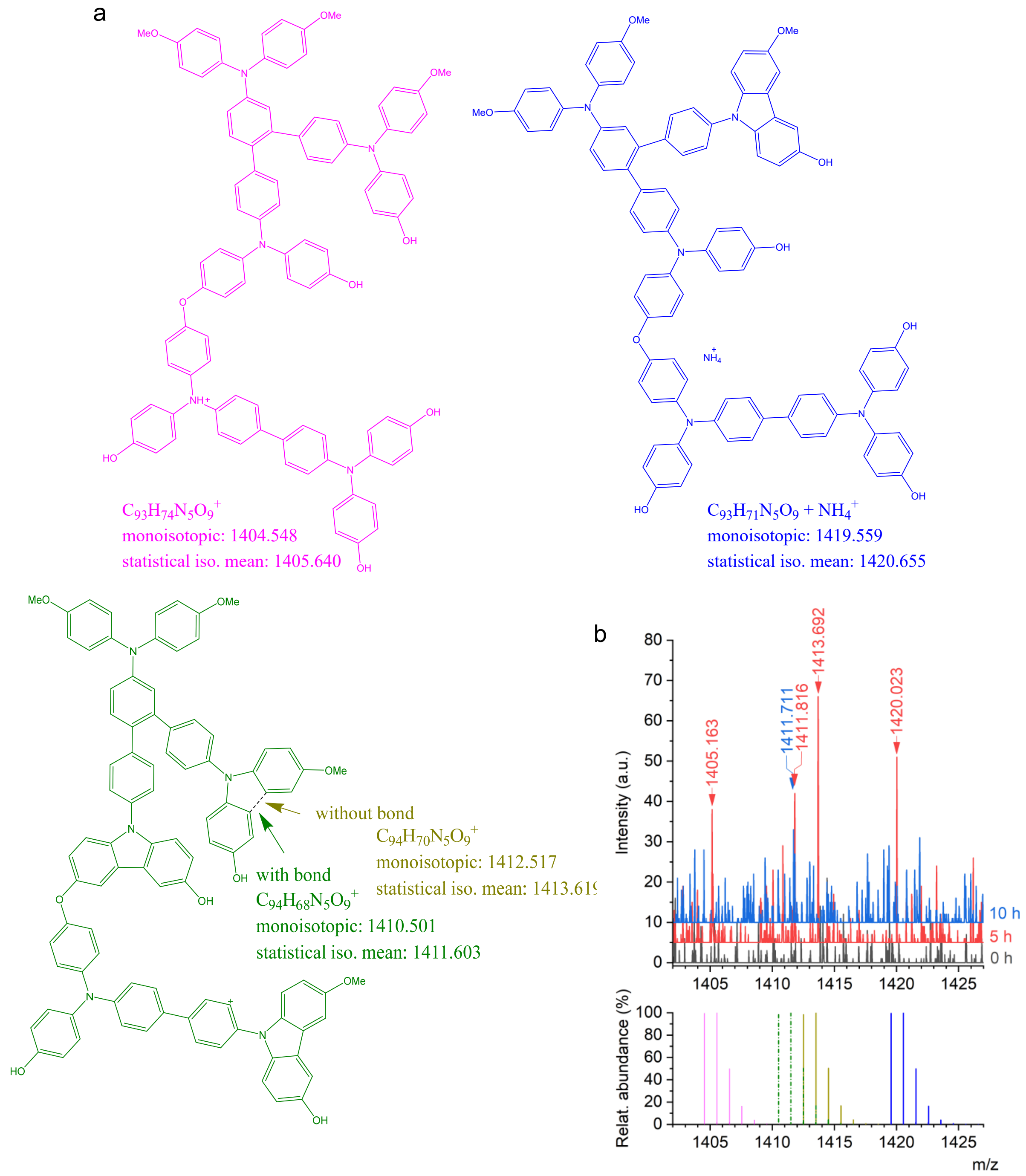

Supplementary Fig. 16-7. MALDI-TOF analysis of chemical structures. Chemical structures (**a**) of the cross-reaction products that yield the measured m/z (**b**) of MeO-TPD irradiated by UV for 0, 5 and 10 h, compared to the theoretical isotopic patterns of identified chemical structures shown in (a).

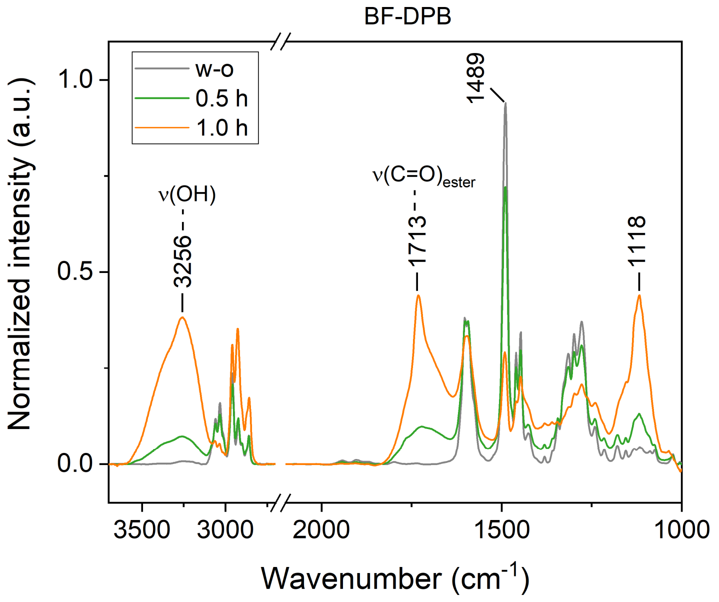

Supplementary Fig. 17. ATR-FTIR spectra of BF-DPB thin films before (40 nm) and after 0.5 h (23 nm) and 1.0 h (5 nm) of UV irradiation. Spectra are normalized to the layer thickness.

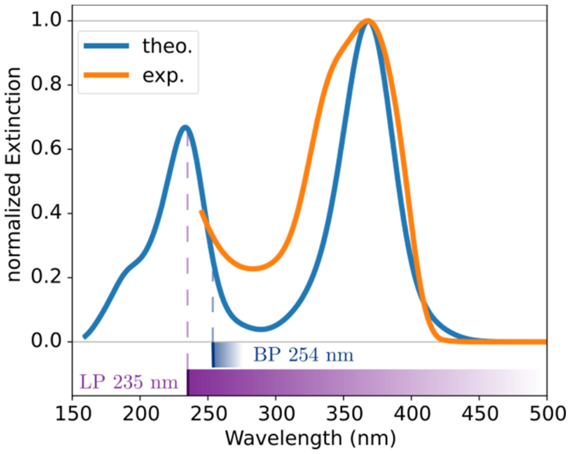

Supplementary Fig. 18-1. Comparison of theoretical GW/BSE and experimental extinction properties of BF-DPB. The extinction coefficient is normalized to the maximum in the considered spectral range.

Theoretical Methods

Ground State Geometry

We employ the density functional theory at the PBE functional[1] level to find the ground state geometry of the BF-DPB gas phase molecule. The calculations are performed with the CP2K software package[2], utilizing Goedecker-Teter-Hutter pseudopotentials[3,4] and TZVP-MOLOPT basis sets from the BASIS_MOLOPT library. A plane-wave cutoff energy of 350 Ha is applied throughout. We relax the BF-DPB molecule until the maximum force is below 0.0001 Ha/a_0 . The resulting equilibrium structure is depicted in **Supplementary Fig. 18-2.**

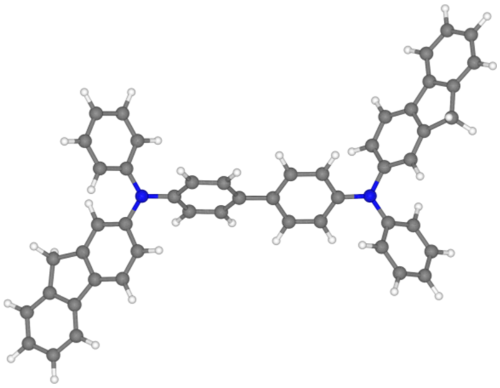

Supplementary Fig. 18-2. Ground state equilibrium structure of the BF-BPD gas phase molecule.

Singlet Excited State Energies and Transition Dipole Moments

To compute the optically active singlet excited state energies and their respective ground-to-excited state transition dipole moments, we use the GW/BSE scheme[5] in combination with the Coulomb-fitting resolution of identity approach[6,7] as implemented in CP2K. Here, we utilize the evGW0 variant in combination with an aug-cc-pVDZ primary basis set and aug-cc-pVDZ-RIFIT as the RI

basis. To prevent potentially severe underestimation of the excited state energies using semi-local functionals[8], we initialize the GW/BSE procedure by long-range corrected hybrid density functional theory (CAM-B3LYP[9]). To make the BSE calculations numerically feasible, we take all Kohn-Sham orbitals that are with ∓ 40 eV around the frontier orbitals into account.

Absorption Spectrum

To compare with the experimental optical extinction spectrum of BF-DPB, we use the spatial average of the diagonal components of the imaginary part of the dipole-dipole susceptibility

$$\underline{\kappa}(\omega) \propto \frac{1}{3}\left[\chi''_{xx}(\omega) + \chi''_{yy}(\omega) + \chi''_{zz}(\omega)\right] .$$

This imaginary part of the susceptibility can be written as,

$$\chi''_{ij}(\omega) = \sum_a d^*_{a,i} d^*_{a,j} \delta(\hbar\omega - E_a + E_0) ,$$

where $d_{\alpha,i}$, $d_{\alpha,j}$ are the transition dipole moments from the ground state to the excited state α in direction $i, j \in \{x, y, z\}$, and E_a, E_0 are the excited and ground state energies accessed by the GW/BSE scheme. We replace the delta-distributions by a normalized Gaussian of the form

$$\delta(\hbar\omega - E_a + E_0) \to exp[-(\omega - E_a + E_0)^2/(2\Gamma(\omega)^2)]/\sqrt{2\pi\Gamma(\omega)^2}$$

with a broadening $\Gamma(\omega) = \gamma\omega$ and $\gamma = 0.045$. We shift the theoretical absorption spectrum by +0.18 eV to align the low-energy band maxima. This modest shift is within the expected errors of the GW/BSE method[8,10]. The resulting normalized spectrum is compared to the experimental one in **Supplementary Fig. 18-1**.

.

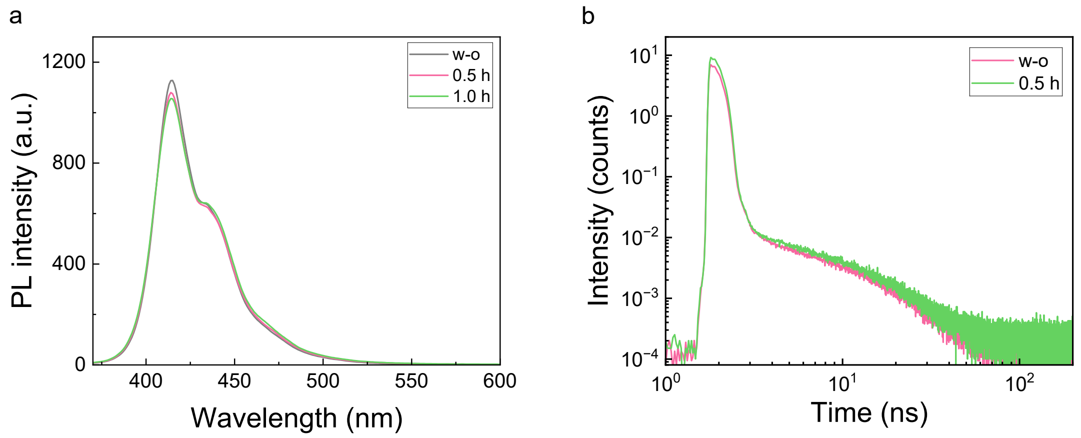

Supplementary Fig. 19. Analysis of UV-induced photoproducts on electronic properties. (**a**) PL and (**b**) TCSPC spectra of BF-DPB:NDP9 (10 wt.%, 100 nm) films under UV irradiation.

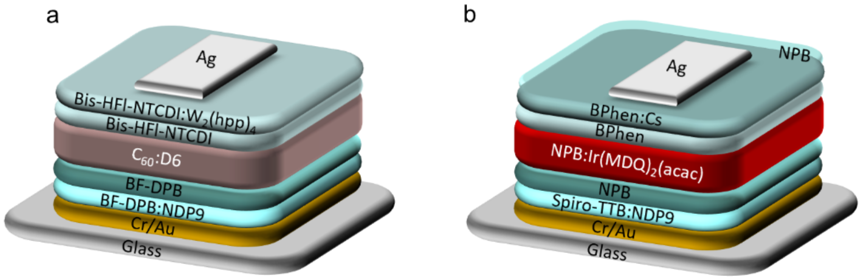

Supplementary Fig. 20. Architectures of UV-irradiated devices. (**a**) OPDs and (**b**) OLEDs**.** The thickness of BF-DPB:NDP9 and Spiro-TTB:NDP9 is changed by UV irradiation for cavity tuning.

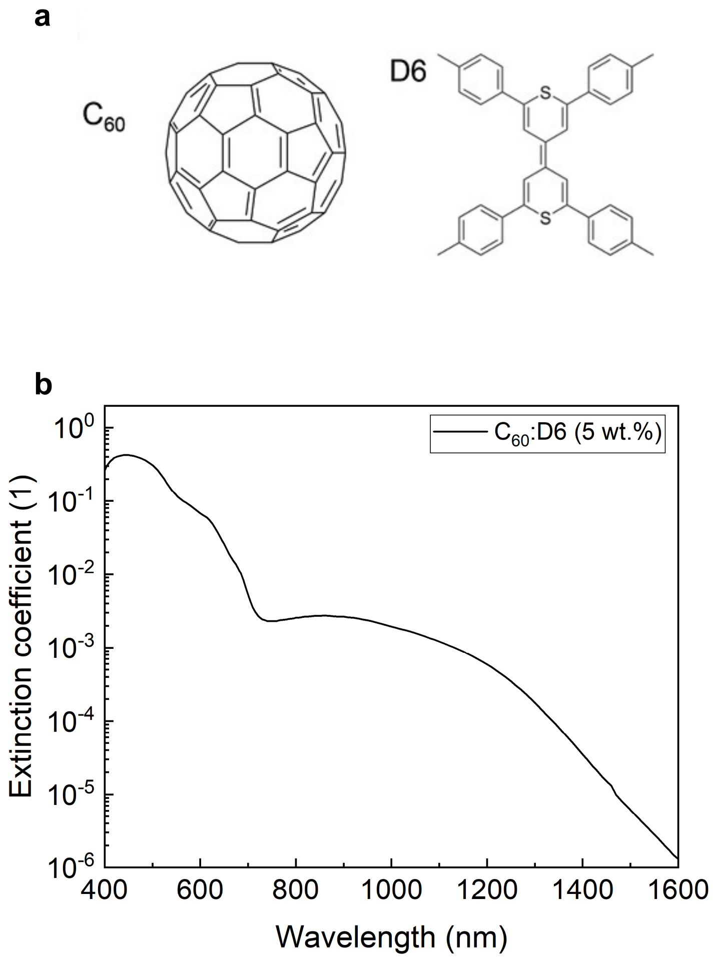

Supplementary Fig. 21. Optical properties of the active layer of UV-irradiated OPDs. (**a**) Molecular structures of C_{60} (acceptor) and D6 (donor). (**b**) Extinction coefficients of C_{60}:D6 (5 wt.%) active blend, derived from the sensitive *EQE* spectrum.

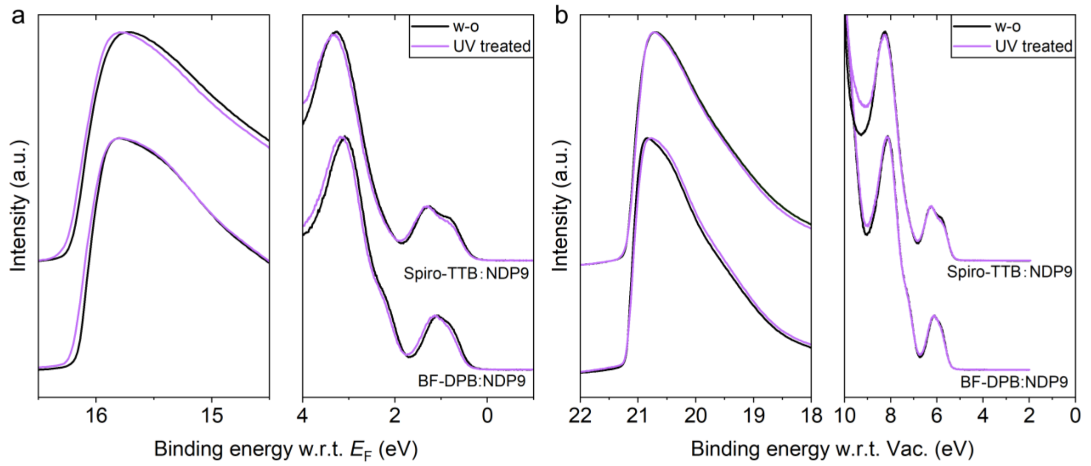

Supplementary Fig. 22. UPS spectra of BF-DPB:NDP9 (10 wt.%) and Spiro-TTB:NDP9 (10 wt.%) before and after 0.5 h of UV treatment. The initial thickness of both films is 40 nm. (**a**) Secondary electron cutoff (SECO) and valence region spectra referenced to the Fermi level (E_F). (**b**) Corresponding spectra referenced to the vacuum level (Vac).

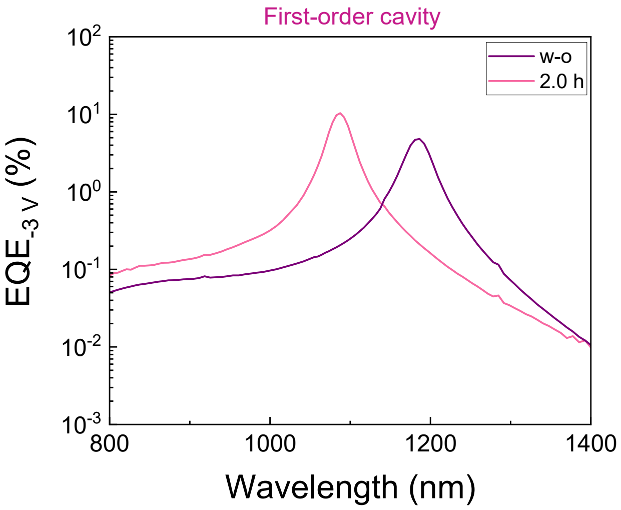

Supplementary Fig. 23. *EQE*$_{\mathbf{PV}}$ spectra of UV-treated OPDs at the first-order cavity under -3 V.

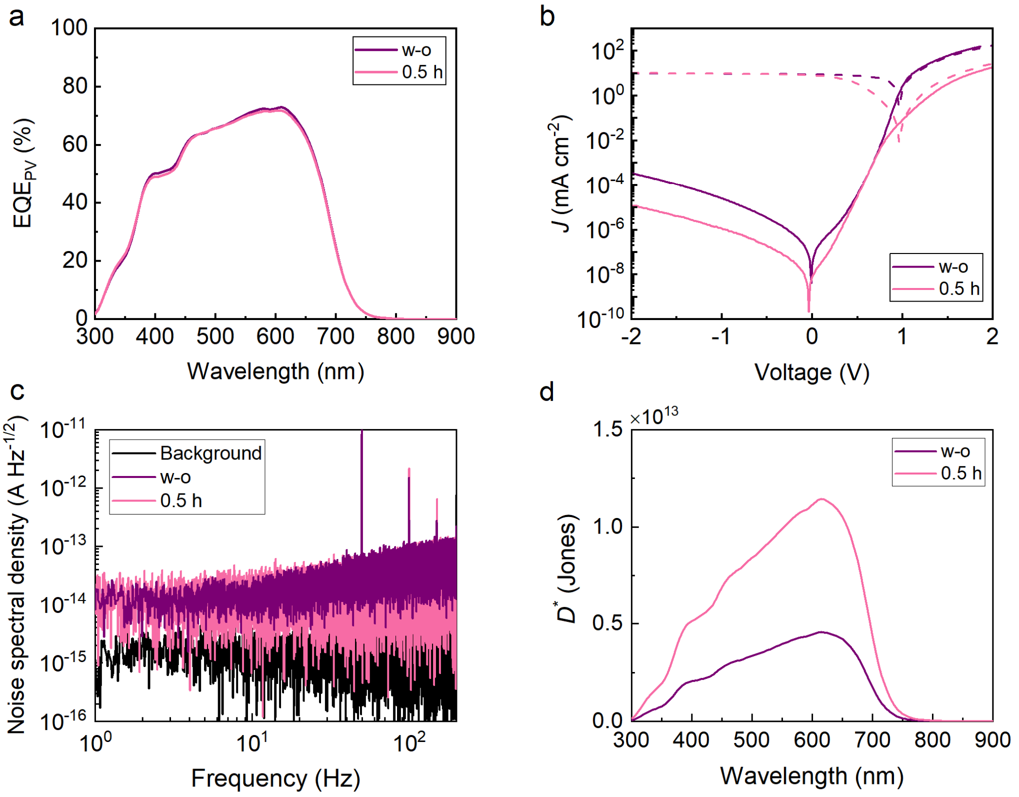

Supplementary Fig. 24. Performance metrics of UV-treated OPDs with a p-i-n architecture. (**a**) EQE_{PV} at 0 V and (**b**) *J-V* characteristics in the light (dashed lines) and dark conditions (solid lines). (**c**) Noise spectral density at 0 V. (**d**) Specific detectivity based on noise measurement at 0 V. The device architecture is ITO / BPAPF:NDP9 (10 wt.%, 40 nm) (under UV treatment) / BPAPF (5 nm) / DCV2-5T-Me(3,3):C_{60} (2:1, 40 nm) / C_{60} (15 nm) / HATNA-Cl_6 (10 nm) / HATNA-Cl_6:$W_2(hpp)_4$ (3 wt.%, 10 nm) / Al (100 nm).

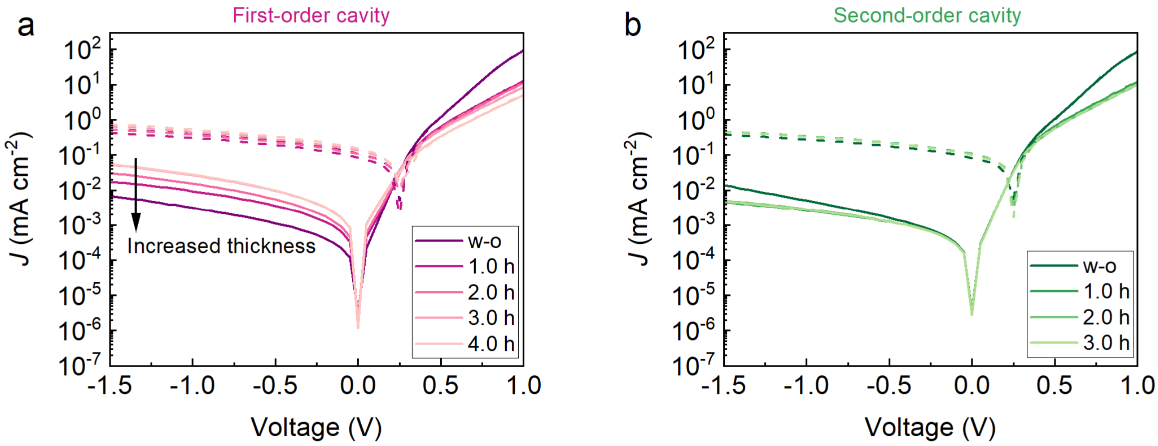

Supplementary Fig. 25. Current density-voltage (*J-V*) characteristics of UV-irradiated OPDs. *J-V* characteristics in the light (dash lines) and dark conditions (solid lines) at the (**a**) first and (**b**) second-order resonances. The light current is measured under the illumination intensity of 1000 W m^{-2}. BF-DPB:NDP9 (10 wt.%) are 110 nm (first-order) and 445 nm (second-order) in the respective OPDs prior to the UV treatment.

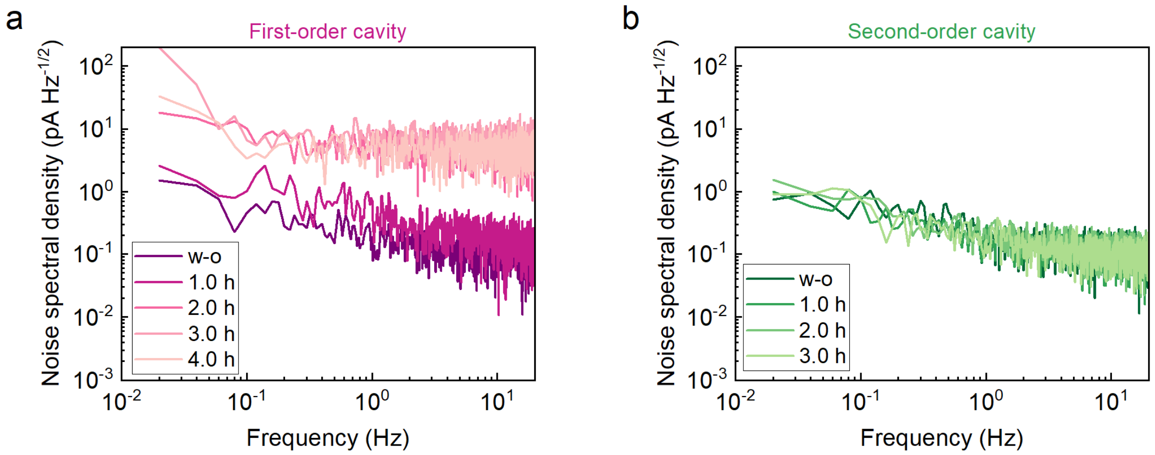

Supplementary Fig. 26. Noise level of UV-irradiated OPDs. Noise spectral density of UV-treated OPDs at the (**a**) first and (**b**) second-order resonances. BF-DPB:NDP9 (10 wt.%) are 110 nm (first-order) and 445 nm (second-order) in the respective OPDs prior to the UV treatment.

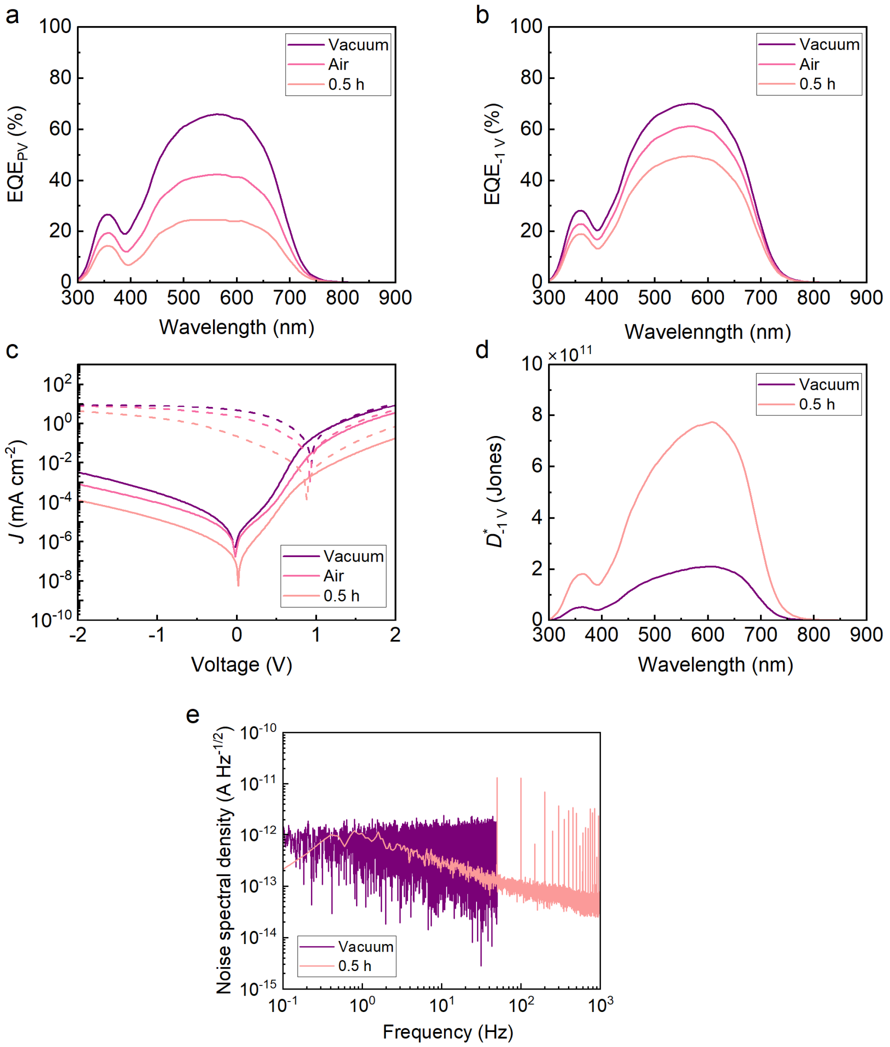

Supplementary Fig. 27. Performance metrics of UV-treated OPDs with an n-i-p architecture. (**a**) EQE_{PV} spectra at 0 V. (**b**) EQE_{PV} spectra under -1 V. (**c**) *J-V* characteristics in the light (dashed lines) and dark conditions (solid lines). (**d**) Specific detectivity at -1 V based on noise measurement. (**e**) Noise spectral density at -1 V. The device architecture is ITO / HATNA-Cl_6 (10 nm) / HATNA-Cl_6:$W_2(hpp)_4$ (3 wt.%, 10 nm) / C_{60} (15 nm) / DCV2-5T-Me(3,3):C_{60} (2:1, 40 nm) / BPAPF (5 nm) / BPAPF:NDP9 (10 wt.%, 40 nm) (under UV treatment) / Al (100 nm).

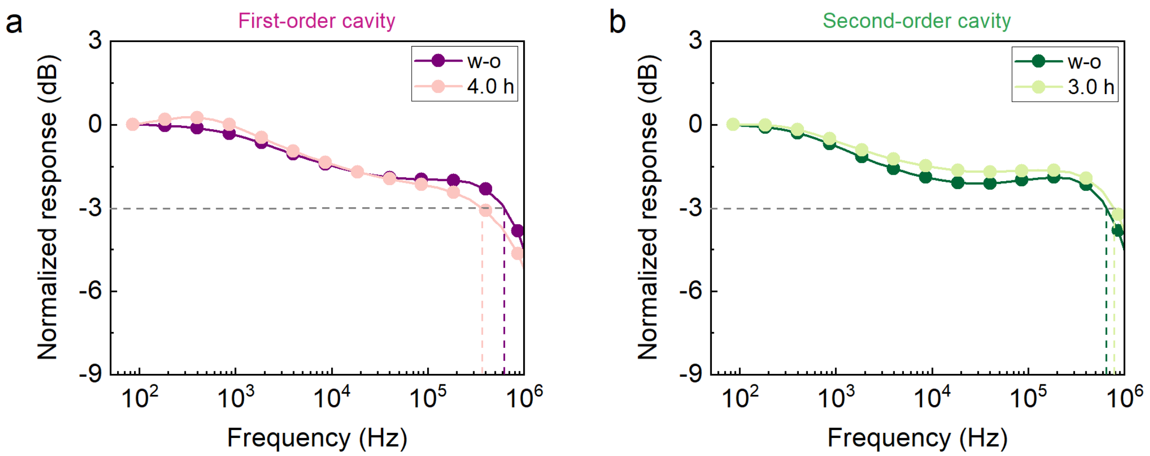

Supplementary Fig. 28. Response speed of UV-irradiated OPDs. Normalized response of UV-irradiated OPDs under at the (**a**) first and (**b**) second-order resonances as a function of input signal frequency (455 nm LED, 3500 W m^{-2}). Vertical dash lines mark the cut-off frequencies obtained at -3 dB. BF-DPB:NDP9 (10 wt.%) are 110 nm (first-order) and 445 nm (second-order) in the respective OPDs prior to the UV treatment.

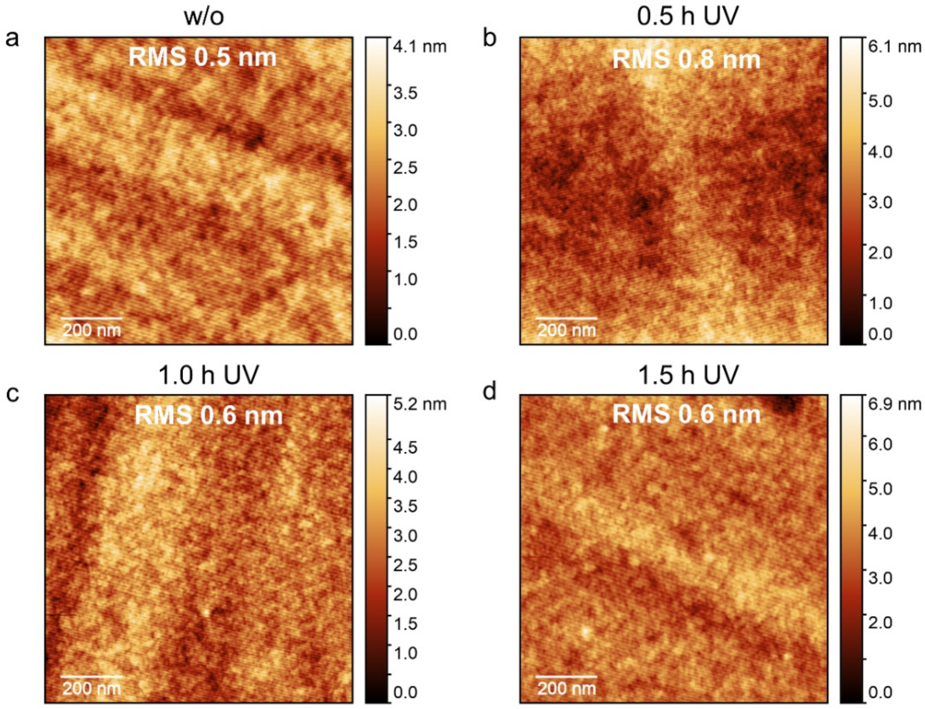

Supplementary Fig. 29. AFM images of Spiro-TTB:NDP9 films (10 wt.%, 100 nm). (**a**) Before and after UV irradiation for (**b**) 0.5 h, (**c**) 1.0 h, and (**d**) 1.5 h.

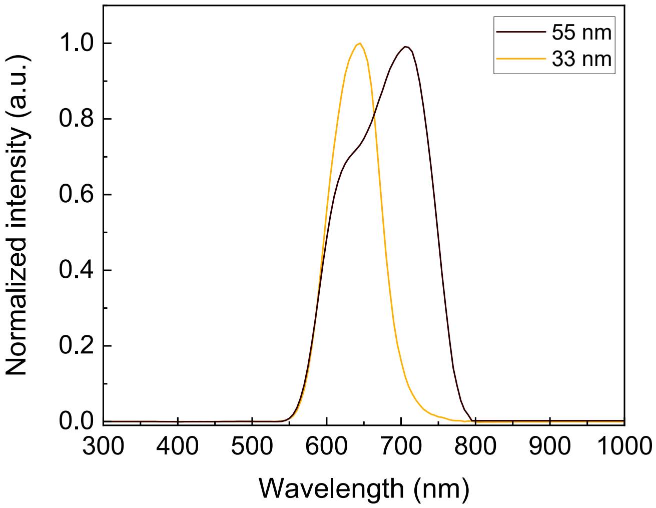

Supplementary Fig. 30. Simulated OLED emission spectra with varying HTL (Spiro-TTB:NDP9) thickness.

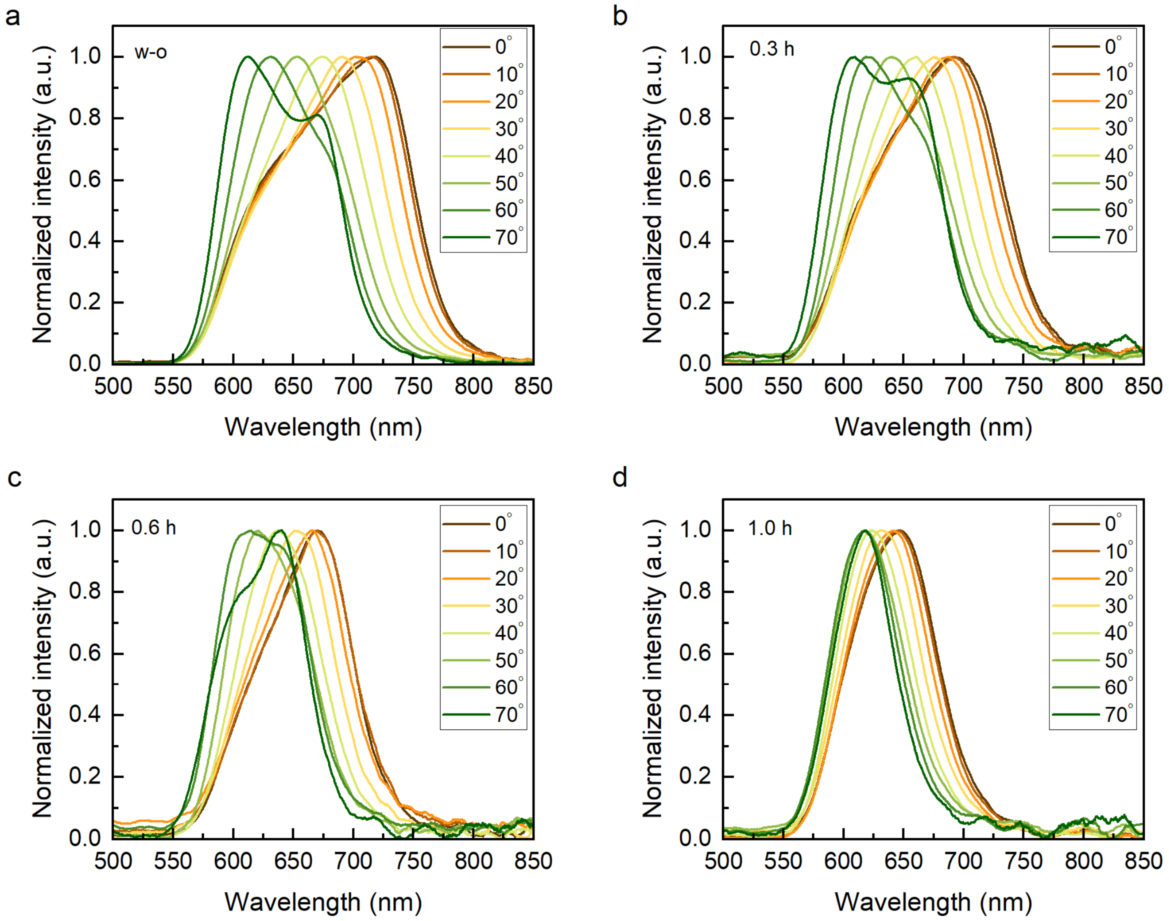

Supplementary Fig. 31. **The angular distribution of the spectral radiant intensity for the cavity red OLEDs.** (**a**) Before and after UV irradiation of (**b**) 0.3 h, (**c**) 0.6 h, and (**d**) 1.0 h.

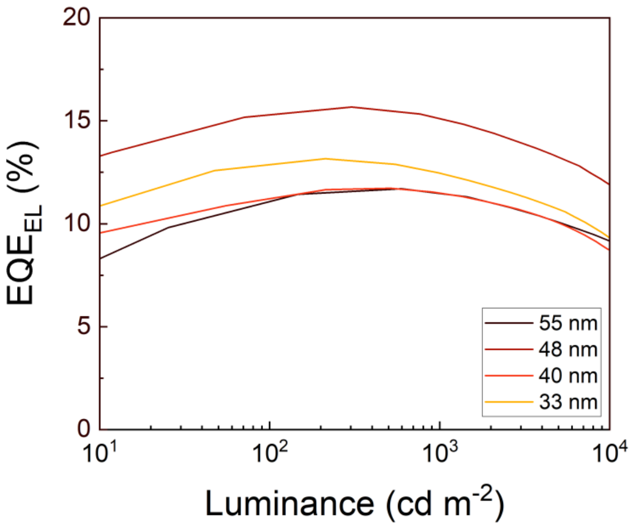

Supplementary Fig. 32. *EQE*EL versus luminance for red OLEDs with physically varied Spiro-TTB:NDP9 (10 wt.%) thicknesses.

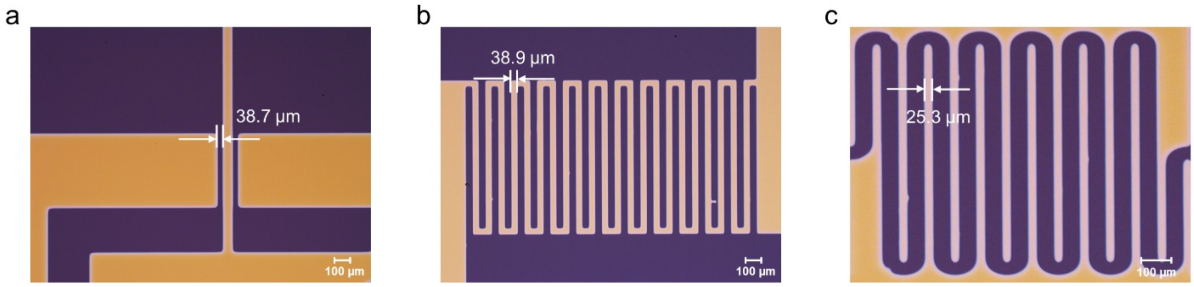

Supplementary Fig. 33. Micrometer-scale organic patterns induced by UV irradiation. Resolutions of organic patterns after UV treatment with (**a**, **b**) linear edges and (**c**) curved edges. The designed lateral resolutions of the structures are (**a**) 40 µm, (**b**) 40 µm, and (**c**) 25 µm.

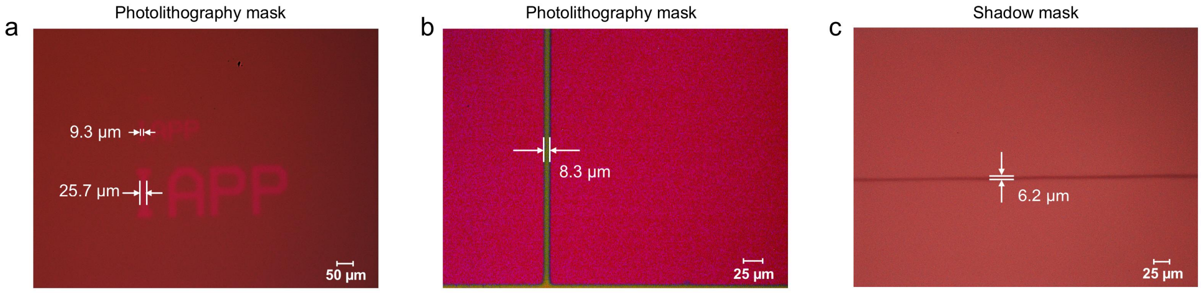

Supplementary Fig. 34. UV-induced organic patterning with sub-10 µm resolution. Resolutions of organic patterns after UV treatment with (**a**, **b**) photolithography masks, and (**c**) shadow masks. The designed lateral resolutions of the structures are (**a**) 10 µm (top) and 25 µm (bottom), (**b**) 8 µm, and (**c**) 5 µm.

Supplementary Table 1. Thickness reduction of BF-DPB and BF-DPB:NDP9 films under UV irradiation. The thickness values are obtained from ellipsometry measurements.

Initial thickness ~ 280 nm					
	**Irradiation time (h)**	**Irradiation energy density (10^6 J m^{-2})**	**Thickness (nm)**	**Reduction rate (nm h^{-1})**	**Reduction rate (10^4 J m^{-2} nm^{-1})**
**BF-DPB**	0	0	285.6	24.8 ± 0.9	3.5 ± 0.1
	1	0.9	270.2		
	2	1.8	244.0		
	3	2.7	209.8		
	4	3.6	178.7		
	6	5.4	129.7		
	8	7.2	78.1		
	12	10.8	0.0		
**BF-DPB:NDP9 (10 wt.%)**	0	0	260.9	21.6 ± 0.6	4.2 ± 0.1
	1	0.9	247.3		
	2	1.8	226.8		
	3	2.7	201.9		
	4	3.6	182.9		
	6	5.4	144.0		
	8	7.2	103.8		
	12	10.8	0.0		

Supplementary Table 2. Device stacks of investigated OPDs and OLEDs. For the devices, the following materials are used: the electrode materials: Cr (Testbourne Ltd., UK), Au (m&k GmbH, Germany) and Ag (m&k GmbH, Germany), the donor material: D6 (TU Dresden, Germany), the acceptor material C_{60} (Nano-C, USA), the emitter $Ir(MDQ)_2(acac)$ (Lumtec Corp., Taiwan), the transport layer materials: BF-DPB (TCI Deutschland GmbH, Germany), Bis-HFl-NTCDI (Nuremberg Institute of Technology, Germany), Spiro-TTB (Lumtec Corp., Taiwan), NPB (Lumtec Corp., Taiwan), and BPhen (Lumtec Corp., Taiwan), and the dopants: NDP9 (Novaled GmbH, Germany), $W_2(hpp)_4$ (TU Dresden, Germany) and Cs (SAES Getters S.p.A., Italy). Layers under UV irradiation are highlighted in bold.

**OPDs**	
**Devices (A:D)**	**Stack**
C_{60}:D6 (first-order cavity)	**Glass (1 mm) / Cr (3 nm) / Au (100 nm) / BF-DPB:NDP9 (10 wt.%, 110 nm)** / BF-DPB (5 nm) / C_{60}:D6 (5 wt.%, 75 nm) / Bis-HFl-NTCDI (5 nm) / Bis-HFl-NTCDI:$W_2(hpp)_4$ (5 wt.%, 90 nm) / Ag (30 nm)
C_{60}:D6 (second-order cavity)	**Glass (1 mm) / Cr (3 nm) / Au (100 nm) / BF-DPB:NDP9 (10 wt.%, 445 nm)** / BF-DPB (5 nm) / C_{60}:D6 (5 wt.%, 75 nm) / Bis-HFl-NTCDI (5 nm) / Bis-HFl-NTCDI:$W_2(hpp)_4$ (5 wt.%, 105 nm) / Ag (30 nm)

**OLEDs**	
**Emission layer**	**Stack**
NPB: $Ir(MDQ)_2(acac)$ (first-order cavity)	**Glass (1 mm) / Cr (3 nm) / Au (80 nm) / Spiro-TTB:NDP9 (7 wt.%, 55 nm)** / NPB (10 nm) / NPB: $Ir(MDQ)_2(acac)$ (10 wt.%, 20 nm) / BPhen (10 nm) / BPhen:Cs (1:1, 65 nm) / Au (2 nm) / Ag (19 nm) / NPB (82 nm)

Supplementary Table 3. Key parameters of UV-irradiated OPDs. Response peak, responsivity (R), thermal noise limit (I_{therm}), measured noise spectral density (I_{real}) with a sample interval of 5 ms, thermal noise limited specific detectivity (D^*_{therm}) and measured specific detectivity (D^*_{real}) at zero bias.

First order cavities							
**HTL (nm)**	**UV treatment (h)**	**Response peak (nm)**	$\boldsymbol{R}$ **(mA W^{-1})**	$\boldsymbol{D^*_{\text{therm}}}$ **(×10^{10} Jones)**	$\boldsymbol{D^*_{\text{real}}}$	$\boldsymbol{I_{\text{therm}}}$ **(10^{-13} A Hz$^{-1/2}$)**	$\boldsymbol{I_{\text{real}}}$
BF-DPB:NDP9 (10 wt.%, initial thickness at 110 nm)	0	1185	6.96	3.01	2.24	0.59	0.79
	1	1155	9.70	2.75	2.05	0.90	1.20
	2	1085	10.40	2.52	0.13	1.05	21
	3	1015	11.89	2.15	0.14	1.41	21
	4	945	11.81	2.15	0.14	1.39	21

Second order cavities							
**HTL (nm)**	**UV treatment (h)**	**Response peak (nm)**	$\boldsymbol{R}$ **(mA W^{-1})**	$\boldsymbol{D^*_{\text{therm}}}$ **(×10^{10} Jones)**	$\boldsymbol{D^*_{\text{real}}}$	$\boldsymbol{I_{\text{therm}}}$ **(10^{-14} A Hz$^{-1/2}$)**	$\boldsymbol{I_{\text{real}}}$
BF-DPB:NDP9 (10 wt.%, initial thickness at 445 nm)	0	1170	1.18	0.43	0.37	6.90	8.06
	1	1140	1.98	0.75	0.62	6.74	8.06
	2	1110	2.55	0.96	0.80	6.73	8.06
	3	1075	3.36	1.28	1.06	6.67	8.06

Supplementary References

1 Perdew, J. P., Burke, K. & Ernzerhof, M. Generalized gradient approximation made simple. *Phys. Rev. Lett.* **77**, 3865 (1996).

2 Kühne, T. D. *et al.* CP2K: An electronic structure and molecular dynamics software package-Quickstep: efficient and accurate electronic structure calculations. *J. Chem. Phys.* **152** (2020).

3 Goedecker, S., Teter, M. & Hutter, J. Separable dual-space Gaussian pseudopotentials. *Phys. Rev. B* **54**, 1703 (1996).

4 Hartwigsen, C., Gœdecker, S. & Hutter, J. Relativistic separable dual-space Gaussian pseudopotentials from H to Rn. *Phys. Rev. B* **58**, 3641 (1998).

5 Blase, X., Duchemin, I. & Jacquemin, D. The Bethe–Salpeter equation in chemistry: relations with TD-DFT, applications and challenges. *Chem. Soc. Rev.* **47**, 1022-1043 (2018).

6 Vahtras, O., Almlöf, J. & Feyereisen, M. Integral approximations for LCAO-SCF calculations. *Chem. Phys. Lett.* **213**, 514-518 (1993).

7 Ren, X. *et al.* Resolution-of-identity approach to Hartree–Fock, hybrid density functionals, RPA, MP2 and GW with numeric atom-centered orbital basis functions. *New J. Phys.* **14**, 053020 (2012).

8 Jacquemin, D., Duchemin, I. & Blase, X. Benchmarking the Bethe–Salpeter formalism on a standard organic molecular set. *J. Chem. Theory Comput.* **11**, 3290-3304 (2015).

9 Yanai, T., Tew, D. P. & Handy, N. C. A new hybrid exchange–correlation functional using the Coulomb-attenuating method (CAM-B3LYP). *Chem. Phys. Lett.* **393**, 51-57 (2004).

10 Bruneval, F., Hamed, S. M. & Neaton, J. B. A systematic benchmark of the ab initio Bethe-Salpeter equation approach for low-lying optical excitations of small organic molecules. *J. Chem. Phys.* **142** (2015).
